# Meroterpenoids and Fucoxanthin from the Brown Seaweed *Sargassum incisifolium*: Solid Lipid Nanoparticle Delivery, Physicochemical Characterization, and Antimicrobial Activity

**DOI:** 10.3390/molecules31101646

**Published:** 2026-05-13

**Authors:** Witness Sibiya, Mogammad L. Samsodien, Jo-Marie Vreulink, Marilize Le Roes-Hill, John J. Bolton, Denzil R. Beukes, Edith Antunes

**Affiliations:** 1 Department of Medical BioSciences, University of the Western Cape, Bellville 7535, South Africa; 4493945@myuwc.ac.za; 2 Department of Chemistry, University of the Western Cape, Bellville 7535, South Africa; 3573207@myuwc.ac.za; 3 Applied Microbial and Health Biotechnology Institute, Cape Peninsula University of Technology, Bellville 7535, South Africa; vreulinkj@cput.ac.za (J.-M.V.); leroesm@cput.ac.za (M.L.R.-H.); 4 Department of Biological Sciences, University of Cape Town, Rondebosch 7701, South Africa; john.bolton@uct.ac.za; 5 School of Pharmacy, University of the Western Cape, Bellville 7535, South Africa

**Keywords:** *Sargassum incisifolium*, marine natural products, meroterpenoids, nanoencapsulation, drug delivery, antimicrobial activity

## Abstract

Marine macroalgae are a rich source of bioactive natural products, although the application of many lipophilic compounds is limited by poor aqueous solubility and instability. This study investigated metabolites isolated from the South African brown seaweed *Sargassum incisifolium* and evaluated a solid lipid nanoparticle (SLN) system to improve their physicochemical properties and enable bioactivity studies. Five metabolites, including one previously unreported derivative and four known metabolites (including fucoxanthin), were isolated and characterized using standard chromatographic and spectroscopic techniques. SLNs composed of stearic acid and Poloxamer 188 were prepared via hot homogenization and characterized using dynamic light scattering, scanning electron microscopy, thermogravimetric analysis, and NMR, which confirmed the efficient encapsulation of the lipophilic compounds. Antimicrobial activity against clinically relevant bacterial and fungal pathogens was evaluated using a resazurin-based microdilution assay, with results expressed as percentage growth relative to untreated controls. The pure compounds exhibited moderate, concentration-dependent activity, while the SLN formulations improved dispersibility, and in several cases, reduced % growth or produced more consistent responses, particularly against Gram-positive bacteria and *Candida auris*. Although activity remained lower than that of conventional antimicrobials, these findings demonstrate that SLN-based delivery enables functional evaluation of hydrophobic marine metabolites and supports further development of *Sargassum*-derived natural products.

## 1. Introduction

The escalation of antimicrobial resistance (AMR) worldwide represents one of the most formidable challenges in modern medicine. Pathogens such as *Staphylococcus aureus* and *Escherichia coli*, along with emerging multi-drug-resistant fungi such as *Candida auris* and *Candida albicans*, are considered a high priority due to the increasingly limited availability of effective treatments.

Marine macroalgae are a prolific source of unique secondary metabolites with potent biological activity [1,2,3,4,5]. Among these, the brown algal genus *Sargassum* is known to produce meroterpenoids containing a terpenoid chain attached to a hydroquinone or quinone moiety, as well as the carotenoid fucoxanthin [6]. These quinone-containing metabolites are of particular interest due to their redox-active properties, which are associated with a range of biological functions, including antimicrobial, antioxidant, and cytotoxic activities [7,8]. Studies on the *Sargassum* species have shown that such compounds can exert antibacterial effects, particularly against Gram-positive organisms, potentially through membrane disruption and interference with redox homeostasis [7,8].

**Figure 1:**
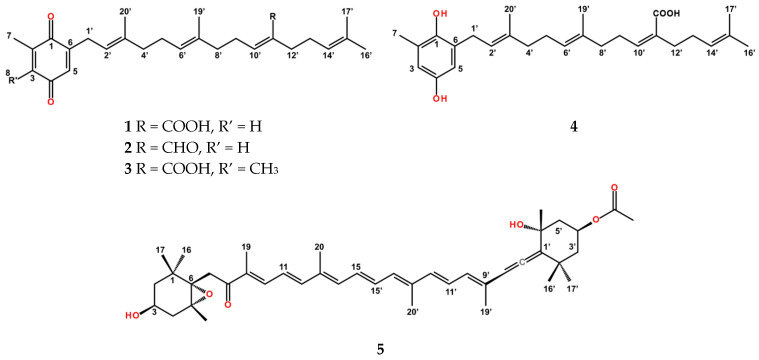
Structures of the compounds used in this study: sargaquinoic acid (**1**), sargaquinal (**2**), 3-methyl sargaquinoic acid (**3**), sargahydroquinoic acid (**4**), and fucoxanthin (**5**).

*Sargassum incisifolium* (previously *Saragassum heterophyllum*) has been shown to produce a range of tetraprenylated toluquinone and toluhydroquinone metabolites. Previous work within our research group established that these compounds have significant inhibitory effects against the malaria parasite *Plasmodium falciparum* [6], with emerging structure–activity relationships suggesting that the redox state plays a critical role in determining biological function. In particular, hydroquinone derivatives tend to display stronger antioxidant activity, while the corresponding quinone and napthoquinone analogues exhibit enhanced antiplasmodial selectivity, indicating that oxidative state and electronic structure are key to the bioactivity displayed [6,9]. Additionally, the presence of a free carboxylic acid functionality appears to be important for maintaining the observed bioactivity and selectivity within this class of compounds [9]. Despite these promising findings, the pharmacological characterization of *S. incisifolium* metabolites remains limited. Only a small number of studies have reported the bioactivity of isolated compounds and there is currently no definitive mechanistic understanding of their antimicrobial or other biological activity. Furthermore, toxicological evaluation is lacking, and few studies have systematically assessed cytotoxicity or selectivity indices, which is a limitation for further therapeutic development. Other studies on *Sargassum* species have identified additional non-polar metabolites, including sterols and glycolipids [10]; however, these investigations are often carried out on crude extracts, making it difficult to attribute biological activity to specific compounds. Despite their strong bioactivity, the utility of the prenylated meroterpenoids (**1**–**4**, Figure 1) and fucoxanthin (**5**, Figure 1) is limited by their lipophilicity, poor aqueous solubility, and bioavailability. Fucoxanthin and the prenylated meroterpenoids are also prone to degradation upon exposure to heat, light, oxygen, and acidic pH, which further limits their dispersion and bioavailability [11,12]. To address these limitations, we utilized Solid Lipid Nanoparticles (SLNs) as a delivery platform. SLNs provide a biocompatible lipid matrix that stabilizes hydrophobic compounds, protects them from oxidative degradation, and facilitates their interaction with microbial cell walls [13,14,15]. SLNs have been widely explored as delivery systems for poorly water-soluble drugs, including anticancer agents such as paclitaxel, as well as antimicrobial agents such as rifampicin, to improve their stability, bioavailability, and therapeutic efficacy [14,16].

In this study, we evaluated the antimicrobial activity of meroterpenoid- and fucoxanthin-loaded SLNs isolated from *Sargassum incisifolium*, against a panel of bacterial and fungal pathogens, with the aim of improving the functional evaluation and delivery of these lipophilic marine natural products.

## 2. Results

### 2.1. Isolation and Characterization of Meroterpenoids and Fucoxanthin

*S. incisifolium* was collected by hand from Noordhoek in the Autumn of 2025. The CH_2_Cl_2_/MeOH extracts were subjected to chromatography to give the initial fractions which were then further purified to give the known compounds sargaquinoic acid (**1**, Fr 3b), sargaquinal (**2**, Fr 3a), sargahydroquinoic acid (**4**, Fr 4), fucoxanthin (**5**, Fr 6b), and the new derivative 3-methyl sargaquinoic acid (**3**, Fr 3c). Further attempts to purify compound **4** led to the re-isolation of compound **1**, and compound **4** was thus used as is.

The spectroscopic data for known compounds are in agreement with previously reported values [6,17,18,19] and are included here in abbreviated form to confirm compound identity and facilitate comparison with the newly identified derivative. The ^13^C NMR spectrum for compound **1** revealed 27 carbon signals, which, in combination with the multiplicity edited HSQC data, allowed the assignment of five methyl, seven methylene, twelve olefinic, and three carbonyl resonances for **1**. The two carbon resonances at δ_C_ 188.0 and 188.1 are characteristic of the presence of a benzoquinone ring, while the third carbonyl carbon signal at δ_C_ 172.2 was attributed to the carboxylic acid group. All spectroscopic data of **1** (Table 1, Figures S1 and S2) were in agreement with literature data reported for sargaquinoic acid (Figure 1) [17].

The ^1^H and ^13^C spectra (Figures S3 and S4) for compound **2** were similar to those of compound **1** except for the exchange of the signal at δ_C_ 172.2 for a new resonance at δ_C_ 195.1 in the ^13^C spectrum as well as a singlet at δ_H_ 9.55 in the ^1^H NMR spectrum, indicative of an aldehyde moiety in **2**. All spectroscopic data for compound **2** (Figure 1, Table 1) agree with that reported by [6].

The NMR spectra for compound **3** showed the typical olefinic side chain signals consistent with a prenylated structure but revealed a slightly modified aromatic substitution pattern to compound **1** as suggested by the lone singlet at δ_H_ 6.46 (Figure S5, Table 1). A change in the chemical shift of C3 from δ_C_ 133.2 in **1** to δ_C_ 140.6 in **3** (Table 1) and an additional methyl resonance at δ_C_ 12.4 (C8) were also observed in the ^13^C spectrum (Figure S6) obtained for compound **3**. The HR-ESI MS data for compound **3** (Figure S7) revealed a molecular ion at *m*/*z* 437.2692 corresponding to a molecular formula of C_28_H_37_O_4,_ confirming the presence of an additional carbon atom. The structure and substitution pattern of the benzoquinone ring for compound **3** were confirmed by 2D NMR data (Table S1, Figures S8–S10), particularly the HMBC data obtained (Figure S10). HMBC correlations were observed between the methyl signal at δ_H_ 1.99 (H_3_-7) to carbons at δ_C_ 187.9 (C1) and 141.0 (C2), and the new methyl signal at δ_H_ 2.02 (H_3_-8) to δ_C_ 141.0 (C2) and 187.7 (C4). Additional HMBC correlations for the methylene signal at δ_H_ 3.12 (H_2_-1′) to δ_C_ 141.0 (C2), 187.7 (C4), 132.0 (C5), 118.2 (C2′) and 139.5 (C3′) and from the lone aromatic methine (H5) at δ_H_ 6.46 to C3 (δ_C_ 140.6), C4 (δ_C_ 187.7) and C1′ (δ_C_ 27.4) established connectivity between the aromatic core and prenyl chain. The placement of the additional methyl on the benzoquinone was thus confirmed by the change in the chemical shift of C3 (and to a lesser extent C2) from δ_C_ 133.2 to 140.6, as well as the observation of a NOESY correlation from H1′ to H5, which further confirmed the placement of the methyl C8 at position C3. The chemical shifts for the side chain moiety remained largely unchanged. Comparison with literature data for SQA (**1**) thus confirmed that compound **3** is a new derivative, rather than an oxidation artifact, and the spectroscopic data, together with the HR-ESI MS data, support the assignment of compound **3** as 3-methyl sargaquinoic acid.

The absence of the benzoquinone carbonyl resonances at δ_C_ 188.0 and 188.1, together with the appearance of two phenolic resonances at δ_C_ 146.3 and 148.8 in the ^13^C spectrum (Table 1), suggested that compound **4** is the hydroquinoic derivative of **1**. Analysis of the spectroscopic data for compound **4** (Figure 1, Figures S11 and S12, Table 1) and comparison with literature data confirmed **4** to be sargahydroquinoic acid [18].

The 1D NMR spectra obtained for fucoxanthin are shown in Figures S13 and S14. The ^13^C spectrum (Figure S14) for the bright orange-red pigment **5** (Figure 1) revealed 42 resonances, which were identical to those reported for fucoxanthin [19].

### 2.2. Synthesis and Characterization of Encapsulated Solid Lipid Nanoparticles

To address the limitations of the isolated lipophilic metabolites, including poor aqueous solubility and instability, SLNs were prepared using a stearic acid–Poloxamer 188 system via hot homogenization. The resulting SLNs exhibited particle sizes ranging from 141 to 186 nm with relatively low polydispersity indices, indicating uniform particle size distribution (Table 2, Figure S15) [20]. Zeta potential values between −29 and −25 mV suggested good colloidal stability. Importantly, particle sizes within this nanometer range are considered favourable for biological interaction, as nanoparticles in the 100–200 nm size range are more likely to interact efficiently with microbial cell surfaces and may enhance local concentration at the membrane interface [21].

After 14 days, an increase in particle size was observed for most formulations, which could arise from slow lipid core relaxation or trace Ostwald ripening [20]. After 14 days, the polydispersity index (PDI) remained largely monodisperse, while the zeta potential remained strongly negative, suggesting partial aggregation over time but the overall retention of colloidal stability [20,22].

The stability of the SLN formulations was evaluated in biologically relevant media (nutrient broth, tryptic soy broth, and yeast malt) over 24 h using DLS (Table S2). The nanoparticles maintained sizes within the nanometer range across all media, with a slight increase in hydrodynamic radii over 24 h. Particle sizes increased from 194 to 209 nm at t = 0 to 233–270 nm at 24 h. This indicates moderate aggregation or swelling in complex media [23]. The PDI remained low (<0.17), suggesting that the formulations retained uniform size distribution. The zeta potential values became more negative over time (from −6 to −14 mV), which reflects the interactions between the nanoparticle surface and components of the growth media.

These physicochemical characteristics are therefore consistent with observed biological performance i.e., nanoscale particle size and surface charge are expected to enhance dispersion in aqueous environments and promote interaction with microbial cell surfaces, particularly in Gram-positive bacteria where the absence of an outer membrane facilitates closer contact with nanoparticle formulations [21]. This is likely to contribute to the improved and more consistently antimicrobial activity observed for the SLN-encapsulated compounds compared to their free counterparts.

Scanning electron microscopy (SEM) analysis confirmed the formation of discrete, approximately spherical particles (Figure S16), while the thermogravimetric analysis (Figure S17) indicated improved thermal stability of the encapsulated systems relative to the free compounds.

**Figure 2:**
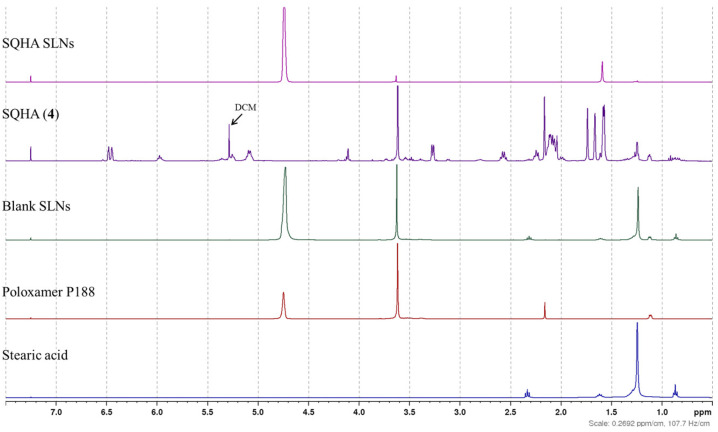
^1^H NMR spectra (400 MHz, CDCl_3_) of Stearic acid (blue), Poloxamer 188 (red), blank solid lipid nanoparticles (Blank SLNs, green), Sargahydroquinoic acid alone (SQHA (**4**), purple), and Sargahydroquinoic acid-loaded solid lipid nanoparticles (SQHA SLNs, pink) showing the incorporation of the components into the SLN formulation.

To confirm the incorporation of the bioactive metabolites into the SLN formulations, ^1^H NMR analyses were performed on both the supernatant and extracted nanoparticle fractions. Following centrifugation of the SLN dispersion (10,000 rpm, 40 min), the supernatant was extracted into CDCl_3_ and analyzed (Figure 2). The signals corresponding to the SQHA (**4**, Figure 2), SQA (**1**), the crude extract (Figure S18), or fucoxanthin (**5**, Figure S19) were observed to be below the detection limit, indicating that these compounds were not present in the external aqueous phase and were effectively retained within the nanoparticle matrix.

In a complementary experiment, the metabolite-loaded SLNs were subjected to exhaustive extraction with dichloromethane, after which the combined organic phases were dried and reconstituted in CDCl_3_ for NMR analyses. These spectra clearly revealed the presence of the crude extract components, SQHA, and fucoxanthin, for the crude extract-, SQHA- and fucoxanthin-loaded SLNs, confirming the successful incorporation into the SLNs. This forced extraction approach provides strong evidence for the efficient encapsulation of the lipophilic metabolites within the lipid nanoparticle system.

### 2.3. Antimicrobial Activity of the Extract, Compounds **1**, **4** and **5**, and the Encapsulated SLNs

**Figure 3:**
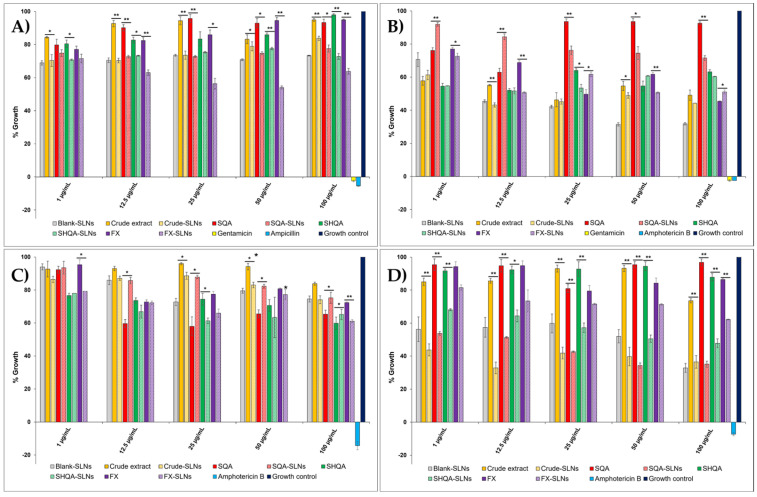
Antimicrobial and antifungal activity of *Sargassum incisifolium* crude extract, isolated compounds (SQA (**1**), SQHA (**4**), and FX (**5**), and their corresponding SLN formulations against (**A**) *Escherichia coli* ATCC 25922 (nutrient broth), (**B**) *Staphylococcus aureus* ATCC 33591 (tryptic soy broth), (**C**) *Candida albicans* ATCC 90028 (yeast malt medium), and (**D**) *Candida auris* ATCC MYA-5001 (yeast malt medium), determined using a resazurin-based microdilution assay at concentrations ranging from 1 to 100 µg/mL. Percentage resazurin reduction is expressed relative to the untreated growth control (100%). Standard antimicrobial agents (gentamicin and ampicillin as antibacterials, and amphotericin B as antifungal) were included as positive controls. Blank SLNs were used to assess carrier effects. Data are presented as mean ± SD (*n* = 3). Statistical comparisons between free compounds and corresponding SLN formulations were performed using paired two-tailed t-tests. * *p* < 0.05, ** *p* < 0.01.

The antimicrobial activity of the crude extract, isolated compounds (SQA (**1**), SQHA (**4**), and fucoxanthin (FX, **5**)), and their SLN formulations was evaluated using a resazurin-based microdilution assay against representative clinically relevant organisms associated with antimicrobial resistance (Figure 3). SQA (**1**), SQHA (**4**), fucoxanthin (**5**), and the crude extract were selected for formulation and antimicrobial evaluation based on their biological relevance, structural diversity, and prior evidence highlighting the importance of the carboxylic acid functionality in controlling bioactivity [9]. Other isolated compounds were not included due to limited availability. The selected panel included the representative bacterial (*Escherichia coli*, *Staphylococcus aureus*) and fungal (*Candida albicans*, and *Candida auris*) pathogens. Antimicrobial activity is expressed as percentage growth relative to the untreated control (100%), where lower % growth values indicate greater antimicrobial effect. The SLNs demonstrated acceptable stability in biological media over 24 h (Table S2), which supports their suitability for antimicrobial evaluation.

Across all organisms, the crude extract and isolated compounds showed moderate, concentration-dependent effects on microbial growth over the tested range (1–100 μg/mL) (Figure 3). The positive controls (gentamicin and ampicillin for bacteria, amphotericin B for fungi) showed near-complete suppression of growth, while the untreated controls confirmed full viability. Blank SLNs showed negligible effects.

For *E. coli* (Figure 3A), relatively high % growth values (>60%) were observed for all samples, indicating limited antibacterial activity against this Gram-negative organism. In contrast, *S. aureus* (Figure 3B) exhibited greater sensitivity to the tested compounds, with SQA (**1**) exhibiting comparatively stronger activity (lowest % growth values) at several concentrations. This is consistent with previous studies on structurally related quinone-containing metabolites isolated from *Sargassum* species, which preferentially inhibit Gram-positive bacteria [24]. This behaviour is in line with literature reports that the outer membrane of Gram-negative bacteria such as *E. coli* imposes a significant permeability barrier to hydrophobic molecules, while Gram-positive bacteria lack this barrier, therefore presenting a greater opportunity for lipophilic compound interaction with the cell wall and cytoplasmic membrane. In addition, quinone-based compounds have been reported to disrupt bacterial membranes and redox homeostasis, which may further contribute to their antimicrobial effects [7]. For both bacterial strains, SLN formulations generally resulted in lower or more consistent % growth values compared to the corresponding free compounds, although this varied with concentration.

Interestingly, non-linear dose-dependent behaviour was observed for several samples, where lower concentrations produced comparable or slightly greater growth suppression than intermediate concentrations. This phenomenon is consistent with hermetic or biphasic responses reported for natural products and may be due to complex interactions between components within the crude extracts or dose-dependent variations of microbial responses [25,26,27,28].

Encapsulation of the compounds into SLNs generally resulted in reduced % growth (i.e., greater growth suppression) or more consistent effects compared to the free compounds (Figure 3). However, this trend was not uniform across all systems. In particular, for SQA (**1**) in the antifungal assay (Figure 3C), the SLN formulation did not consistently enhance activity relative to the free compound, and in some cases showed slightly higher % growth values. This may reflect differences in release kinetics, compound–matrix interactions, or the intrinsic activity of the free compound, which may already be sufficiently bioavailable under the assay conditions. Where enhancement was observed, this may arise from improved aqueous dispersibility, sustained release from the lipid matrix, and enhanced interaction of nanoparticles with microbial cell surfaces. Nanoparticle-based delivery systems have been shown to increase contact time at biological interfaces and promote uptake or membrane interaction, thereby improving the apparent activity of encapsulated lipophilic compounds [29,30].

In the antifungal assays, both *C. albicans* (Figure 3C) and *C. auris* (Figure 3D) displayed moderate susceptibility to the tested samples. For *C. albicans*, SQA (**1**) and SQHA (**4**) demonstrated reduced % growth relative to the crude extract at intermediate concentrations, while SLN formulations showed variable effects depending on the compound and concentration. In contrast, FX-SLNs showed reduced % growth at selected concentrations compared to the free fucoxanthin. Similar trends were observed for *C. auris*, where compound- and concentration-dependent differences were evident between the free and encapsulated samples. Notably, the SLN formulations resulted in substantially lower % growth values (approximately 40%) compared to the corresponding free compounds, which remained at ~85% growth across several concentrations. This reduction indicates that nanoencapsulation significantly enhances the antimicrobial effect against *C. auris*, suggesting improved delivery and/or interaction of the encapsulated compounds with this organism. These observations were further supported by statistical analysis. Fucoxanthin exhibited moderate antibacterial activity, in agreement with previous reports demonstrating stronger activity against Gram-positive bacteria than Gram-negative strains [31]. Fucoxanthin differs structurally from the quinone-containing meroterpenoids, suggesting distinct mechanisms of action that may contribute differently to the overall activity profile observed.

Statistical analysis using paired two-tailed t-tests confirmed that several differences between free compounds and their SLN formulations were statistically significant (* *p* < 0.05, ** *p* < 0.01), particularly at specific concentrations (Figure 3). However, these effects were not uniform across all compounds or organisms, indicating that the influence of encapsulation on antimicrobial activity is both compound- and assay-dependent.

These findings indicate that the tested meroterpenoids and fucoxanthin from *S. incisifolium* exhibit moderate antimicrobial activity, with variability across organisms and concentrations, and that SLN encapsulation can modulate microbial growth responses in a compound-specific manner. While the observed activity is moderate relative to commercial antimicrobial agents, the data shows the value of nanocarrier systems in enabling the functional evaluation and potential application of hydrophobic marine natural products.

## 3. Discussion

The present study expands the chemical and pharmacological understanding of *Sargassum incisifolium* through the isolation of five metabolites including the previously unreported derivative 3-methyl sargaquinoic acid (**3**). The identification of this compound contributes to the growing body of evidence that *Sargassum* species produce structurally diverse quinone-type meroterpenoids with notable biological activity. Previous work on *S. incisifolium* (formerly *S. heterophylum*), has demonstrated that tetraprenylated toluquinones and toluhydroquinones metabolites exhibit significant antiplasmodial activity against *Plasmodium falciparum* [6], while subsequent semi-synthetic studies further highlighted the importance of structural modification and redox state in modulating biological activity [9]. In addition, hydroquinone derivatives such as sargahydroquinoic acid (SQHA, **4**) have shown anti-inflammatory activity through macrophage modulation effects without significant cytotoxicity [8].

In the present study, the isolated compounds exhibited moderate antimicrobial activity, expressed as reductions in % growth relative to untreated controls. This is consistent with previous reports on *Sargassum*-derived meroterpenoids and related quinone metabolites. Quinone-containing compounds have been reported to preferentially inhibit Gram-positive bacteria, likely due to their ability to interact with bacterial membranes and participate in redox cycling processes [24]. The comparatively stronger activity observed against *Staphylococcus aureus* relative to *Escherichia coli* in this study supports this trend and may be attributed to the permeability barrier imposed by the outer membrane of Gram-negative bacteria, which limits the uptake of hydrophobic compounds. This behaviour is also consistent with other studies on fucoxanthin (**5**) [24] and related marine natural products, which demonstrate enhanced activity against Gram-positive organisms.

From a structure–activity perspective, the results agree with previously reported trends for *Sargassum* meroterpenoids. In particular, the redox state of the quinone/hydroquinone system plays a key role in biological function. Hydroquinone derivatives are typically associated with stronger antioxidant and anti-inflammatory activity, while the corresponding quinone analogues exhibit enhanced biological selectivity in systems such as antiplasmodial assays [8,9]. Although the present study does not establish a definitive structure–activity relationship for antimicrobial activity, the comparable behaviour of the isolated compounds suggests that stable electronic and functional group differences influence their biological response.

A key contribution of this study is the demonstration that solid lipid nanoparticle (SLN) formations can be used to enable the biological evaluation of lipophilic marine natural products. Previous studies on *Sargassum* species have largely relied on crude extracts, limiting the ability to assess compound-level bioactivity in aqueous biological systems [10]. In the present study, NMR analyses confirmed the successful incorporation of both the isolated metabolites and crude extract into SLN formulations. The resulting nanoparticles exhibited nanoscale particle sizes (141–186 nm), low polydispersity, and negative zeta potentials, indicating good colloidal stability for biological applications.

Encapsulation within the SLNs influenced antimicrobial behaviour in a compound and concentration-dependent manner. In several cases, SLN formulations resulted in reduced % growth or more consistent responses compared to the corresponding free compounds, particularly against Gram-positive organisms. Statistical analysis supported these observations, with significant differences (* *p* < 0.05, ** *p* < 0.01) observed at specific concentrations. These effects may be attributed to increased aqueous dispersibility, sustained release from the lipid matrix, and potentially enhanced interaction between nanoparticles and microbial membranes, potentially increasing the local concentration of the bioactive compounds at the site of action. Such behaviour is consistent with previous reports demonstrating that lipid-based nanocarriers can improve the apparent activity of hydrophobic antimicrobial agents [14].

However, this effect was not universally observed. For example, in the antifungal assay against *C. albicans* (Figure 3C), SQA-SLN formulations did not consistently reduce % growth relative to the free compound and, in some cases, resulted in slightly higher % growth values. The reduction in % growth observed for *C. auris* upon SLN encapsulation (to ~40% compared to ~85% for the free compounds) (Figure 3D) indicates an improved antimicrobial response, which may be attributed to enhanced delivery and/or interaction of the encapsulated metabolites with the fungal cells; this trend was supported by statistically significant differences (*p* < 0.05, *p* < 0.01) at selected concentrations. This data suggests that factors such as release kinetics, compound–matrix interactions, or the intrinsic activity of the free compound under the assay conditions may influence the overall response. These data show that encapsulation does not uniformly enhance biological activity and must be considered in a compound-specific scenario.

Despite showing some promise, several limitations need to be acknowledged. The antimicrobial activity observed was moderate compared to conventional antibiotics and antifungal agents, indicating that these compounds in their current form are unlikely to function as stand-alone therapeutics. In addition, stability studies indicated short-term aggregation in biological media, which suggests the need for further formulation optimization. Furthermore, the lack of cytotoxicity and selectivity data limits the ability to assess the therapeutic potential of these compounds, representing an important area for further investigation.

Future work should therefore focus on optimizing the SLN composition to improve long-term stability and delivery performance, as well as assessing cytotoxicity, selectivity, and activity against antibiofilm-forming activity. In addition, combination strategies with established antimicrobial agents may provide synergistic effects and enhance overall efficacy. This work therefore highlights both the opportunities and challenges of *Sargassum*-derived meroterpenoids and demonstrates the importance of formulation strategies in enabling the biological evaluation and future development of lipophilic marine natural products.

## 4. Materials and Methods

### 4.1. General Experimental Procedures

Column chromatography of the seaweed extract was performed on silica gel 60 (0.040–0.063 mm) from Merck KGaA (Darmstadt, Germany). Normal phase thin-layer chromatography (TLC) was performed on Silica gel 60 F254 aluminium sheets purchased from Merck KGaA (Darmstadt, Germany) and visualized under UV light at 254 and 365 nm. NMR samples were prepared in deuterated solvents, and all experiments were performed using a Bruker Avance III HD 400 MHz spectrometer (Rheinstetten, Germany) equipped with a 5 mm BBI probe at 298 K. Chemical shifts were referenced to deuterated solvent peaks (CDCl_3_ δ_H_ 7.25, δ_C_ 77.00) and reported in ppm. Homogenisation and sonication of the SLNs were accomplished using an IKA^®^ T18 digital Ultra Turrax^®^ homogenizer (Staufen, Germany) and Bandelin Sonoplus HD 2070 (Berlin, Germany), respectively. Dynamic light scattering (DLS) studies, Zeta potential (ζ) stability, and particle size distributions of the SLNs were determined using a Malvern Zetasizer Nano ZS instrument (Malvern Instruments Ltd., Worcestershire, UK). All data were processed using Malvern Zeta Sizer software (version 8.00.4813), while statistical analyses were carried out using Excel GraphPad Prism 10.6.1 (GraphPad Software, San Diego, CA, USA). TGA measurements were performed using a Perkin-Elmer TGA 4000 instrument (Stamford, CT, USA). Samples were heated from 30 °C to 850 °C at 10 °C/min under a nitrogen flow rate of 20 mL/minute. Samples were first dried to remove residual solvent, and approximately 2–4 mg of each sample was accurately weighed for TGA analysis. The data was collected and analyzed using Pyris software (version 11.0.3). High-resolution images were obtained using a Zeiss MERLIN Field Emission Scanning Electron Microscope (FE-SEM) (Jena, Germany). For SEM analysis, dried samples were mounted onto aluminium stubs using conductive carbon tape and sputter-coated with a thin gold layer to improve conductivity and reduce charging effects prior to imaging. High-resolution LC-MS data were acquired on a Waters Cyclic Select (Milford, MA, USA) coupled to a Waters UPLC using an ESI probe in Positive and Negative ion mode with the cone voltage set to 21 V, at the Central Analytical Facility at Stellenbosch University.

### 4.2. Seaweed Collection and Identification

The brown seaweed *Sargassum incisifolium* was collected at low tide from Noordhoek near Gqeberha (34°2′24.09″ S, 25°38′16.04″ E), South Africa (March 2025). The organism was identified, and a voucher specimen prepared and assigned the specimen number KOS2025-0316-5, which was deposited at the School of Pharmacy, University of the Western Cape (UWC), South Africa. Freshly collected material was stored at −20 °C until extraction of the whole seaweed. All subsequent procedures were conducted under reduced light conditions to minimize degradation of light-sensitive metabolites such as fucoxanthin.

### 4.3. Isolation and Characterization of the Metabolites

The frozen seaweed (225.90 g wet mass) was thawed and rinsed briefly with distilled water (2 L) to remove debris and salts. Sequential solvent extraction was performed using MeOH (2 L) and followed by a 2:1 mixture of DCM:MeOH for 24 h. The combined extracts were then concentrated under reduced pressure using a rotary evaporator at temperatures below 40 °C to prevent degradation to give the crude extract. The extracted seaweed material was then dried to give 36.45 g dry mass. The organic phases were collected and dried to give 19.47 g of crude extract, which was stored in vials covered with foil at −20 °C until further use. Liquid–liquid partitioning using MeOH and DCM (2:1) was carried out using the crude extract to give the respective fractions with varying polarity.

The secondary metabolites were isolated using column chromatography using the method described by Afolayan et al. (2008) [6] with some modifications. One gram of the CH_2_Cl_2_-MeOH crude extract from liquid–liquid partitioning was dissolved in 2 mL of DCM and applied to celite. The solvent was removed to obtain a powder. The resulting sample was then applied as a powder to a silica gel column (10 g, 2.5 × 6 cm) and successively eluted with a stepwise gradient elution starting with 100% hexane (Fr 1, 2.6 mg), 1:9 EtOAc/hexane (Fr 2, 22.8 mg), 2:8 EtOAc/hexane (Fr 3, 94.6 mg), 3:7 EtOAc/hexane (Fr 4, 185.1 mg), 4:6 EtOAc/hexane (Fr 5, 15.9 mg), 6:4 EtOAc/hexane (Fr 6, 24.2 mg), 8:2 EtOAc/hexane (Fr 7, 6.8 mg) and 100% EtOAc (Fr 8, 92.2 mg), followed by a 1:1 EtOAc/MeOH mixture (Fr 9, 130.4 mg) and 100% MeOH (Fr 10, 15.2 mg). All fractions were subjected to ^1^H NMR analyses, with selected fractions, i.e., Fr 3, Fr 4, Fr 5, and Fr 6, found to contain the ^1^H NMR signals of interest. Additional chromatography on the crude extract using the above procedure, followed by further purification of fractions 3–6 gave the known compounds sargaquinoic acid (**1**, Fr 3b, 41.7 mg), sargaquinal (**2**, Fr 3a, 22.5 mg), and the new derivative 3-methyl sargaquinoic acid (**3**, Fr 3c, 28.9 mg) using a mobile phase of 7:3 EtOAc/Hexane on a silica gel column (1 g), collecting 2 mL fractions at a time. Fractions were combined based on their TLC profiles. Similarly, sargahydroquinoic acid (**4**, Fr 4, 63.7 mg) and fucoxanthin (**5**, Fr 6b, 22.5) were applied to a 1 g silica gel column using a mobile phase of 4:6 EtOAc/hexane and 5:5 EtOAc/hexane, respectively. Fractions (2 mL) were collected and combined according to their TLC profiles.

Sargaquinoic acid (**1**): pale yellow oil; NMR data consistent with published data [14] and available in the Supplementary Materials. Yield: 41.7 mg.

Sargaquinal (**2**): pale yellow oil; NMR data consistent with published data [6] and available in the Supplementary Materials. Yield: 22.5 mg.

3-methyl sargaquinoic acid (**3**): pale yellow oil; ^1^H NMR (400 MHz, CDCl_3_) δ 6.46 (H5, s), 1.99 (H7, m), 2.02 (H8, m), 3.12 (H1′, d, *J* =7.1 Hz), 5.14 (H2′, m), 2.05 (H4′, m), 2.11 (H5′, m), 5.10 (H6′, m), 2.07 (H8′, m), 2.57 (H9′, q, *J* = 7.4), 5.98 (H10′, t, *J* = 7.3), 2.25 (H12′, t, *J* = 7.1), 2.10 (H13′, m), 5.08 (H14′, m), 1.68 (H16′, m), 1.58 (H17′, m), 1.60 (H19′, m), 1.61 (H20′, m); ^13^C NMR (100 MHz, CDCl_3_) δ 187.9 (C1), 141.0 (C2), 140.6 (C3), 187.7 (C4), 132.0 (C5), 148.0 (C6), 12.0 (C7), 12.4 (C8), 27.4 (C1′), 118.2 (C2′), 139.5 (C3′), 39.6 (C4′), 27.8 (C5′), 124.5 (C6′), 134.6 (C7′), 39.0 (C8′), 28.2 (C9′), 145.3 (C10′), 130.5 (C11′), 34.6 (C12′), 26.3 (C13′), 123.4 (C14′), 132.2 (C15′), 25.6 (C16′), 17.7 (C17′), 172.1 (C18′), 16.1 (C19′), 15.9 (C20′), HRESI-MS *m*/*z* 437.2692 [M-H]^−^ (calc. for C_28_H_37_O_4_); Yield 28.9 mg.

Sargahydroquinoic acid (**4**): pale yellow oil; NMR data consistent with published data [15] and available in the Supplementary Materials. Yield: 63.7 mg.

Fucoxanthin (**5**): bright orange-red solid; NMR data consistent with published data [16] and available in the Supplementary Materials. Yield: 22.5 mg.

### 4.4. Preparation and Characterization of the Metabolite-Loaded Solid Lipid Nanoparticles

The SLNs were synthesized using a hot homogenization method adapted from the method described by Eskiler et al. (2018) [32] with some modifications. For SLN preparation, the lipid phase consisted of stearic acid (50 mg), which was heated to 75 °C until completely molten. The bioactive compound (25 mg), previously dissolved in dichloromethane (1 mL), was incorporated into the molten lipid under continuous stirring to ensure homogeneous dispersion. The organic solvent was allowed to evaporate during heating to facilitate the formation of a uniform lipid–drug matrix. Separately, the aqueous phase was prepared by dissolving Poloxamer 188 (25 mg) in distilled water and heating the solution to 75 °C to match the temperature of the lipid phase. Maintaining both phases at identical temperatures minimized premature lipid solidification during emulsification. The hot aqueous surfactant solution was then gradually added to the molten lipid phase under mechanical stirring at 1000 rpm for 5 min to form a coarse pre-emulsion. This pre-emulsion was subjected to high-speed homogenization at 10,000 rpm for 5 min to reduce droplet size and improve dispersion uniformity. The resulting hot nano-emulsion was further processed by probe sonication for 1 min using pulse mode to enhance particle size reduction and distribution homogeneity. Immediately after sonication, the formulation was rapidly cooled in an ice bath to induce recrystallization of the lipid matrix, leading to the formation of solid lipid nanoparticles. Blank SLNs were prepared under identical conditions but without the incorporation of any bioactive compound. The final SLN dispersions were diluted in distilled water adjusted to pH 7.4 and stored at 4 °C until further characterization and biological evaluation.

The encapsulation efficiency of the nanoparticles was determined using a quantitative NMR using the ERETIC method. The procedure enabled the direct quantification of the unencapsulated (free) compound present in solution. To evaluate the presence of the free compound (i.e., non-encapsulated metabolites), the SLN formulations were subjected to centrifugation, then organic extraction in CDCl_3_ followed by quantitative ^1^H NMR analysis. An aliquot (500 μL) of the freshly prepared SLN dispersion was transferred to a 2 mL Eppendorf tube, and 1 mL of CDCl_3_ was added. The mixture was vortexed vigorously for 5 min and centrifuged at 10,000 rpm for 40 min at room temperature (25 °C). Centrifugation resulted in the formation of a visible pellet containing the SLNs, while any non-encapsulated lipophilic compound was expected to partition into the CDCl_3_ supernatant. A 500 µL aliquot of the supernatant was carefully removed and transferred into an NMR tube for analysis. Quantitative ^1^H NMR spectra were acquired using a 90° pulse, with 128 scans collected per sample under identical acquisition parameters to ensure reproducibility and accurate signal integration. The ERETIC protocol was then employed to determine the quantity of compounds of interest using 1,4-dinitrobenzene as the external reference. In a complementary experiment, the whole metabolite-loaded SLN was subjected to forced extraction using DCM (10 mL × 3), after which the extracts were dried, and reconstituted using CDCl_3_. ^1^H NMR spectra were again acquired using the same parameters described above.

### 4.5. Antimicrobial Assays

The Gram-negative bacterial strain, *Escherichia coli* ATCC 25922, and the Gram-positive strain, the methicillin-resistant *Staphylococcus aureus* ATCC 33591 (MRSA), were selected for antibacterial evaluation. Fungal strains included *Candida albicans* ATCC 24433, *Candida albicans* ATCC 90028, and *Candida auris* ATCC MYA-5001.

Bacterial strains were grown overnight in their recommended liquid media, tryptic soy broth (TSB) and nutrient broth (NB), respectively, at 37 °C with shaking at 160 rpm. Cultures were adjusted to an optical density of OD_600_ = 0.1, corresponding to approximately 1 × 10^6^ CFU/mL, before use. Fungal strains were streaked from glycerol stocks onto yeast malt (YM) agar plates and incubated at 30 °C or 37 °C for 24 h, depending on strain requirements. Five colonies were suspended in sterile saline and adjusted spectrophotometrically to match a 0.5 McFarland standard (absorbance 0.12–0.15 at 530 nm). The suspensions were diluted in YM broth to yield final inocula of approximately 0.5 × 10^3^ to 2.5 × 10^3^ cells/mL. All inocula were freshly prepared before performing the assays and were tested in triplicate.

Fucoxanthin-, SQA-, SHQA-, extract- and blank-loaded SLN formulations, including the unloaded compounds and crude extracts alone, were reconstituted and diluted in sterile medium to twice the highest test concentration. Two-fold serial dilutions were prepared in broth to generate a final concentration range of 1 µg/mL–100 µg/mL (1 µg/mL, 12.5 µg/mL, 25 µg/mL, 50 µg/mL, and 100 µg/mL). Positive controls consisted of standard antibiotics/antifungals: for bacteria (e.g., gentamicin or ampicillin) and for *Candida* (amphotericin B), with each prepared in the same solvent and medium. A solvent control (sterile broth with 5%, *v*/*v*, DMSO) was included as a negative control, along with a broth-only sterility control. All test dilutions and controls were freshly prepared on the day of assay and tested in triplicate.

Antimicrobial activity against bacterial and fungal strains was evaluated using a resazurin-based broth microdilution assay in sterile 96-well plates. Briefly, 100 µL of each nanoparticle or compound dilution was added per well, followed by 100 µL of standardized microbial inoculum, resulting in a final volume of 200 µL per well. Plates were incubated for 24 h at 37 °C for bacterial strains and appropriate conditions for fungal strains.

Following incubation, 20 µL of freshly prepared resazurin solution (0.001% *w*/*v* in phosphate-buffered saline) was added to each well, and plates were further incubated for 2–4 h. Reduction of resazurin (blue) to resorufin (pink) was used as an indicator of microbial viability. Absorbance was measured at 570 nm and 600 nm using a microplate reader, and the extent of resazurin reduction was used to calculate percentage growth relative to untreated controls.

Antimicrobial activity is expressed as percentage growth relative to the untreated control, where lower % growth values indicate greater antimicrobial effect. All experiments were conducted in triplicate independent assays, and results are presented as mean values ± standard deviation. Data analysis was performed using GraphPad Prism 10.6.1 (GraphPad Software, San Diego, CA, USA). Statistical comparisons between free compounds and corresponding SLN formulations were performed using two-tailed paired Student’s t-tests (*n* = 3), with significance indicated as *p* < 0.05 (*) and *p* < 0.01 (**).

## 5. Conclusions

This study demonstrates that *Sargassum incisifolium* is a valuable source of lipophilic meroterpenoids and expands the known chemical diversity of this species through the identification of a previously unreported derivative. The isolated compounds exhibited moderate antimicrobial activity, with a preference for Gram-positive bacteria, which is consistent with the behaviour of quinone-type metabolites. Importantly, the use of solid lipid nanoparticles enabled the effective evaluation of these hydrophobic compounds in aqueous biological systems and, in several cases, improved the consistency of their antimicrobial response.

While the observed bioactivity remains lower than that of conventional antimicrobial agents, the results show the importance of formulation strategies in enabling the biological assessment of lipophilic marine natural products. The findings also support previously reported relationships between structure, redox state, and biological function in *Sargassum*-derived metabolites.

This work provides a foundation for further investigation of *Sargassum* meroterpenoids as bioactive compounds and demonstrates that nanocarrier-based delivery systems are a valuable tool for advancing their evaluation and potential application.

## Figures and Tables

**Table 1:** Comparison of ^13^C NMR data (100 MHz, CDCl_3_) for compounds **1**–**4**.

Carbon #	1	2	3	4
1	188.1 (C)	188.0 (C)	187.9 (C)	146.3 (C)
2	145.9 (C)	146.0 (C)	141.0 (C)	125.5 (C)
3	133.2 (CH)	133.2 (CH)	140.6 (C)	115.4 (CH)
4	188.0 (C)	188.0 (C)	187.7 (C)	148.8 (C)
5	132.2 (CH)	132.3 (CH)	132.0 (CH)	114.0 (CH)
6	148.5 (C)	148.4 (C)	148.0 (C)	127.6 (C)
7	16.0 (CH_3_)	16.0 (CH_3_)	12.0 (CH_3_)	16.0 (CH_3_)
8	-	-	12.4 (CH_3_)	-
1′	27.5 (CH_2_)	27.5 (CH_2_)	27.4 (CH_2_)	29.9 (CH_2_)
2′	118.0 (CH)	118.1 (CH)	118.2 (CH)	121.7 (CH)
3′	139.8 (C)	139.7 (C)	139.5 (C)	138.1 (C)
4′	39.6 (CH_2_)	39.5 (CH_2_)	39.6 (CH_2_)	39.5 (CH_2_)
5′	26.3 (CH_2_)	26.3 (CH_2_)	27.8 (CH_2_)	26.03 (CH_2_)
6′	124.5 (CH)	125.1 (CH)	124.5 (CH)	124.2 (CH)
7′	134.6 (C)	133.9 (C)	134.6 (C)	134.7 (C)
8′	39.0 (CH_2_)	38.3 (CH_2_)	39.0 (CH_2_)	39.0 (CH_2_)
9′	28.2 (CH_2_)	27.0 (CH_2_)	28.2 (CH_2_)	28.3 (CH_2_)
10′	145.3 (CH)	155.0 (CH)	145.3 (CH)	145.4 (CH)
11′	130.6 (C)	143.2 (C)	130.5 (C)	130.6 (C)
12′	34.6 (CH_2_)	24.3 (CH_2_)	34.6 (CH_2_)	34.5 (CH_2_)
13′	27.9 (CH_2_)	26.3 (CH_2_)	26.3 (CH_2_)	27.8 (CH_2_)
14′	123.5 (CH)	123.6 (CH)	123.4 (CH)	123.4 (CH)
15′	132.2 (C)	133.2 (C)	132.2 (C)	132.2 (C)
16′	25.7 (CH_3_)	25.7 (CH_3_)	25.6 (CH3)	25.6 (CH_3_)
17′	15.9 (CH_3_)	17.6 (CH_3_)	17.7 (CH3)	17.7 (CH_3_)
18′	172.4 (C)	195.2 (C)	172.1 (C)	172.5 (C)
19′	16.1 (CH_3_)	16.0 (CH_3_)	16.1 (CH3)	16.0 (CH_3_)
20′	16.1 (CH_3_)	16.1 (CH_3_)	15.9 (CH3)	16.1 (CH_3_)

# number.

**Table 2:** Dynamic Light Scattering (DLS), hydrodynamic radii (d), and Zeta potential measurements for SLNs immediately after synthesis and 14 days later (in parentheses) for the α-tocopherol (Toco), the crude extract (Crude), sargaquinoic acid (SQA, **1**), sargahydroquinoic acid (SHQA, **4**) and fucoxanthin (FX, **5**)-loaded SLNs.

Formulation	Hydrodynamic Size (d (nm))	Polydispersity Index (PDI)	Zeta Potential (mV)
Blank-SLNs	141.65 ± 0.82 (376.50 ± 7.06)	0.186 (0.191)	−25.9 ± 1.00 (−28.63 ± 1.72)
Toco-SLNs	183.61 ± 8.95 (345.33 ± 4.70)	0.195 (0.205)	−26.10 ± 1.21 (−32.33 ± 2.04)
Crude-SLNs	176.10 ± 14.70 (376.2 ± 6.60)	0.191 (0.214)	−29.07 ± 1.89 (−32.50 ± 1.76)
SQA (**1**)-SLNs	157.42 ± 1.21 (325.50 ± 6.30)	0.164 (0.247)	−25.03 ± 0.38 (−32.87 ± 0.29)
SHQA (**4**)-SLNs	185.60 ± 6.50 (278.5 ± 13.20)	0.087 (0.227)	−27.43 ± 0.42 (−30.27 ± 1.75)
FX (**5**)-SLNs	168.92 ± 7.26 (309.0 ± 5.33)	0.130 (0.203)	−26.8 ± 0.70 (−31.97 ± 1.07)

## Data Availability

All the research data are available in the Supplementary Materials.

## Supplementary Materials

The following supporting information can be downloaded at: https://www.mdpi.com/article/10.3390/molecules31101646/s1, Figure S1. ^1^H NMR spectrum of compound **1** (Sargaquinoic acid) (CDCl_3_, 400 MHz); Figure S2. ^13^C NMR spectrum of compound **1** (Sargaquinoic acid) (CDCl_3_, 100 MHz); Figure S3. 1HNMR spectrum of compound **2** (Sargaquinal) (CDCl_3_, 400 MHz); Figure S4. ^13^C NMR spectrum of compound **2** (Sargaquinal) (CDCl_3_, 400 MHz); Figure S5. ^1^H NMR spectrum of compound **3** (CDCl_3_, 400 MHz); Figure S6. ^13^C NMR spectrum of compound **3** (CDCl_3_, 400 MHz); Figure S7. LC-HRMS data obtained for compound **3** showing A) the total ion chromatogram (TIC) and B) the mass spectrum of the peak eluting at 10.776 min; Figure S8. COSY spectrum (400 MHz, CDCl_3_) of compound **3** (3-methyl sargaquinoic acid); Figure S9. HSQC spectrum (400 MHz, CDCl_3_) of compound **3** (3-methyl sargaquinoic acid); Table S1. ^1^H (400 MHz), ^13^C (100 MHz) NMR and 2D NMR (COSY, HSQC, HMBC) spectroscopic data for compound **3**; Figure S10. HMBC spectrum (400 MHz, CDCl_3_) of compound **3** (3-methyl sargaquinoic acid); Figure S11. ^1^H NMR spectrum of compound **4** (Sargahydroquinoic acid) (CDCl_3_, 400 MHz); Figure S12. ^13^C NMR spectrum of compound **4** (Sargahydroquinoic acid) (CDCl_3_, 100 MHz); Figure S13. ^1^H NMR spectrum of compound **5** (Fucoxanthin) (CDCl_3_, 400 MHz); Figure S14. ^13^C DEPTQ-135 NMR spectrum for compound **5** (Fucoxanthin) (CDCl_3_, 100 MHz); Table S2. Stability of solid lipid nanoparticles (SLNs) in Nutrient Broth (NB) measured at 0, 1, and 24 h using DLS. The table shows changes in particle size, PDI, and zeta potential over time; Figure S15. Dynamic light scattering (DLS) size distribution profiles of solid lipid nanoparticles (SLNs): (a) blank SLNs, (b) tocopherol-loaded SLNs, (c) crude extract-loaded SLNs, (d) sargaquinoic acid (SQA, 1)-loaded SLNs, (e) sargahydroquinoic acid (SHQA, 4)-loaded SLNs, and (f) fucoxanthin (FX, 5)-loaded SLNs. The measurements represent the hydrodynamic diameter distributions of the nanoparticles in aqueous suspension, confirming nanoscale particle sizes and relatively narrow size distributions across all formulations; Figure S16. SEM images and particle size distribution histograms of the SLN formulations for (A) Blank SLNs, (B) Crude-SLNs (S. incisifolium crude extract), and (C) Tocopherol-SLNs. Scale: 50 µm; Figure S17. TGA profiles for α-tocopherol (black), the crude extract (light blue), blank SLNs (red), α-tocopherol-loaded SLNs (green), and crude extract-loaded SLNs (blue); Figure S18. ^1^H NMR spectra (400 MHz, CDCl_3_) of stearic acid, Poloxamer 188, blank SLNs, crude organic extract, and the crude organic extract-loaded SLNs showing the incorporation of the components into the SLN formulation; Figure S19. ^1^H NMR spectra (400 MHz, CDCl_3_) of stearic acid, Poloxamer 188, blank SLNs, fucoxanthin (**5**), and the fucoxanthin (**5**)-loaded SLNs showing the incorporation of the components into the SLN formulation.
