# Supplementary material for: Meroterpenoids and Fucoxanthin from the Brown Seaweed Sargassum incisifolium: Solid Lipid Nanoparticle Delivery, Physicochemical Characterization, and Antimicrobial Activity
Source: Molecules. 2026 May 13;31(10):1646. doi: 10.3390/molecules31101646 (PMC13210362; doi:10.3390/molecules31101646)
Supplement: Supplementary file 1 [file molecules-31-01646-s001.zip › molecules-4284592-supplementary.pdf]

Supplementary information

Meroterpenoids and Fucoxanthin from the brown seaweed *Sargassum incisifolium*: solid lipid nanoparticle delivery, physicochemical characterization, and antimicrobial activity

Witness Sibiya [1], Luqmaan Samsodien [2], Jo-Marie Vreulink [3], Marilize Le Roes-Hill [3], John J. Bolton [4], Denzil R. Beukes [5,*], and Edith Antunes [2,*]

[1] Department of Medical BioSciences, University of the Western Cape, Bellville 7535, South Africa; 4493945@myuwc.ac.za
[2] Department of Chemistry, University of the Western Cape, Bellville 7535, South Africa; 3573207@myuwc.ac.za (LS); ebeukes@uwc.ac.za (EA)
[3] Biocatalysis and Technical Biology Research Group, Institute of Biomedical and Microbial Biotechnology, Cape Peninsula University of Technology, Bellville 7535, South Africa; LeRoesM@cput.ac.za (MLRH); VreulinkJ@cput.ac.za (JMV)
[4] Department of Biological Sciences, University of Cape Town, Rondebosch 7701, South Africa; john.bolton@uct.ac.za
[5] School of Pharmacy, University of the Western Cape, Bellville 7535, South Africa; dbeukes@uwc.ac.za

* Correspondence: ebeukes@uwc.ac.za; dbeukes@uwc.ac.za; Tel.: +27219594020

Table of Contents

Figure S1. ^{1}H NMR spectrum of compound **1** (Sargaquinoic acid) ($CDCl_3$, 400 MHz)........3

Figure S2. ^{13}C NMR spectrum of compound **1** (Sargaquinoic acid) ($CDCl_3$, 100 MHz). 3

Figure S3. ^{1}HNMR spectrum of compound **2** (Sargaquinal) ($CDCl_3$, 400 MHz).......4

Figure S4. ^{13}C NMR spectrum of compound **2** (Sargaquinal) ($CDCl_3$, 400 MHz). 4

Figure S5. ^{1}H NMR spectrum of compound **3** ($CDCl_3$, 400 MHz)........5

Figure S6. ^{13}C NMR spectrum of compound **3** ($CDCl_3$, 400 MHz). 5

Figure S7. LC-HRMS data obtained for compound **3** showing A) the total ion chromatogram (TIC) and B) the mass spectrum of the peak eluting at 10.776 min. .6

Figure S8. COSY spectrum (400 MHz, $CDCl_3$) of compound **3** (3-methyl sargaquinoic acid). 6

Figure S9. HSQC spectrum (400 MHz, $CDCl_3$) of compound **3** (3-methyl sargaquinoic acid). 7

Table S1. ^{1}H (400 MHz), ^{13}C (100 MHz) NMR and 2D NMR (COSY, HSQC, HMBC) spectroscopic data for compound **3**........8

Figure S11. ^{1}H NMR spectrum of compound **4** (Sargahydroquinoic acid) ($CDCl_3$, 400 MHz)........9

Figure S12. ^{13}C NMR spectrum of compound **4** (Sargahydroquinoic acid) ($CDCl_3$, 100 MHz)........9

Figure S13. ^{1}H NMR spectrum of compound **5** (Fucoxanthin) ($CDCl_3$, 400 MHz). 10

Figure S14. ^{13}C DEPTQ-135 NMR spectrum for compound **5** (Fucoxanthin) ($CDCl_3$, 100 MHz)........10

Figure S15. Dynamic light scattering (DLS) size distribution profiles of solid lipid nanoparticles (SLNs): (a) blank SLNs, (b) tocopherol-loaded SLNs, (c) crude extract-loaded SLNs, (d) sargaquinoic acid (SQA, **1**)-loaded SLNs, (e) sargahydroquinoic acid (SHQA, **4**)-loaded SLNs, and (f) fucoxanthin (FX, **5**)-loaded SLNs. The measurements represent the hydrodynamic diameter distributions of the nanoparticles in aqueous suspension, confirming nanoscale particle sizes and relatively narrow size distributions across all formulations.......11

Table S2. Stability of solid lipid nanoparticles (SLNs) in Nutrient Broth (NB) measured at 0, 1, and 24 hours using DLS. The table shows changes in particle size, PDI, and zeta potential over time........11

Figure S16. SEM images and particle size distribution histograms of the SLN formulations for (A) Blank SLNs, (B) Crude-SLNs (*S. incisifolium* crude extract), and (C) Tocopherol-SLNs. Scale: 50 μm........12

Figure S17. TGA profiles for α-tocopherol (black), the crude extract (light blue), blank SLNs (red), and a-tocopherol loaded SLNs (green), and crude extract loaded SLNs (blue)........13

Figure S18. ^{1}H NMR spectra (400 MHz, $CDCl_3$) of stearic acid, Poloxamer P188, blank SLNs, crude organic extract, and the crude organic extract-loaded SLNs showing the incorporation of the components into the SLN formulation........13

Figure S19. ^{1}H NMR spectra (400 MHz, $CDCl_3$) of stearic acid, Poloxamer P188, blank SLNs, fucoxanthin (**5**), and the fucoxanthin (**5**)-loaded SLNs showing the incorporation of the components into the SLN formulation........14

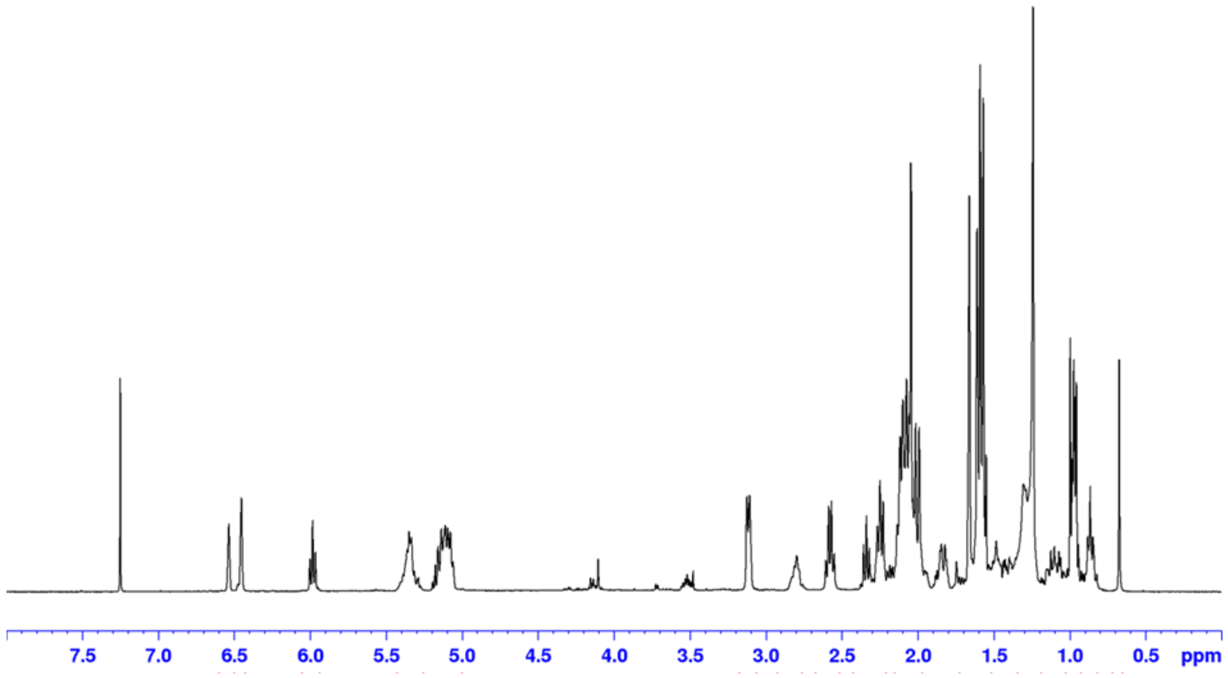

Figure S1. ^{1}H NMR spectrum of compound **1** (Sargaquinoic acid) ($CDCl_3$, 400 MHz).

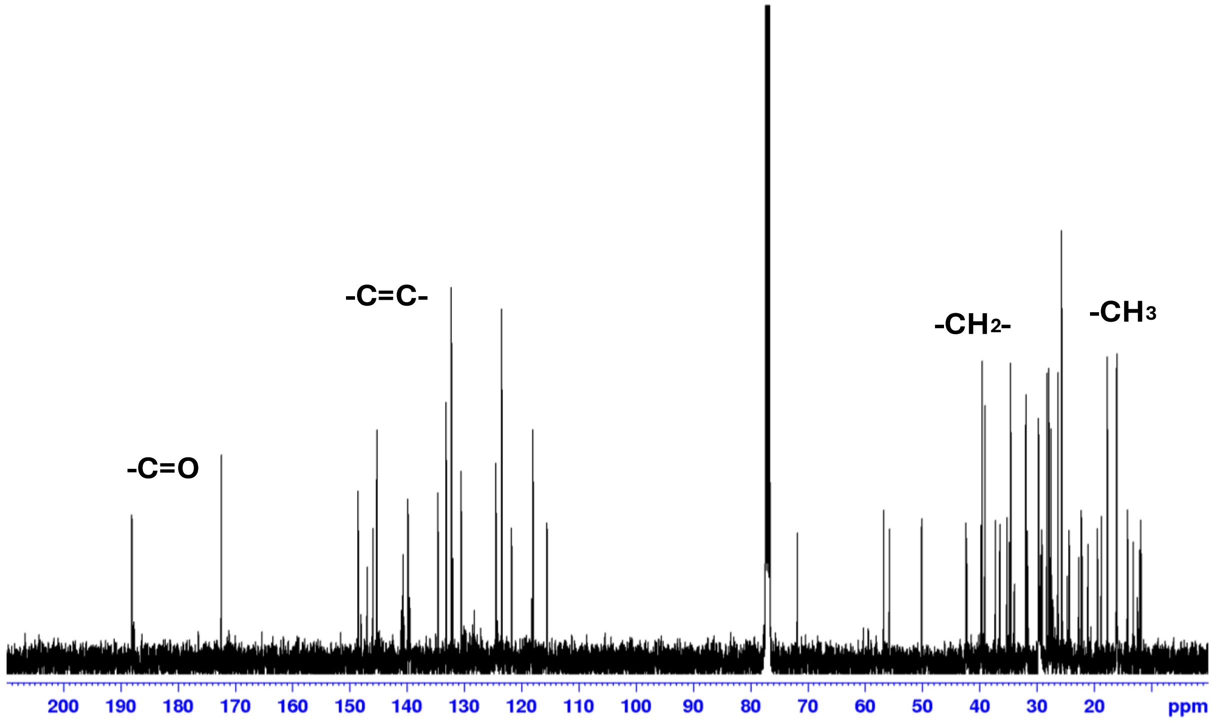

Figure S2. ^{13}C NMR spectrum of compound **1** (Sargaquinoic acid) ($CDCl_3$, 100 MHz).

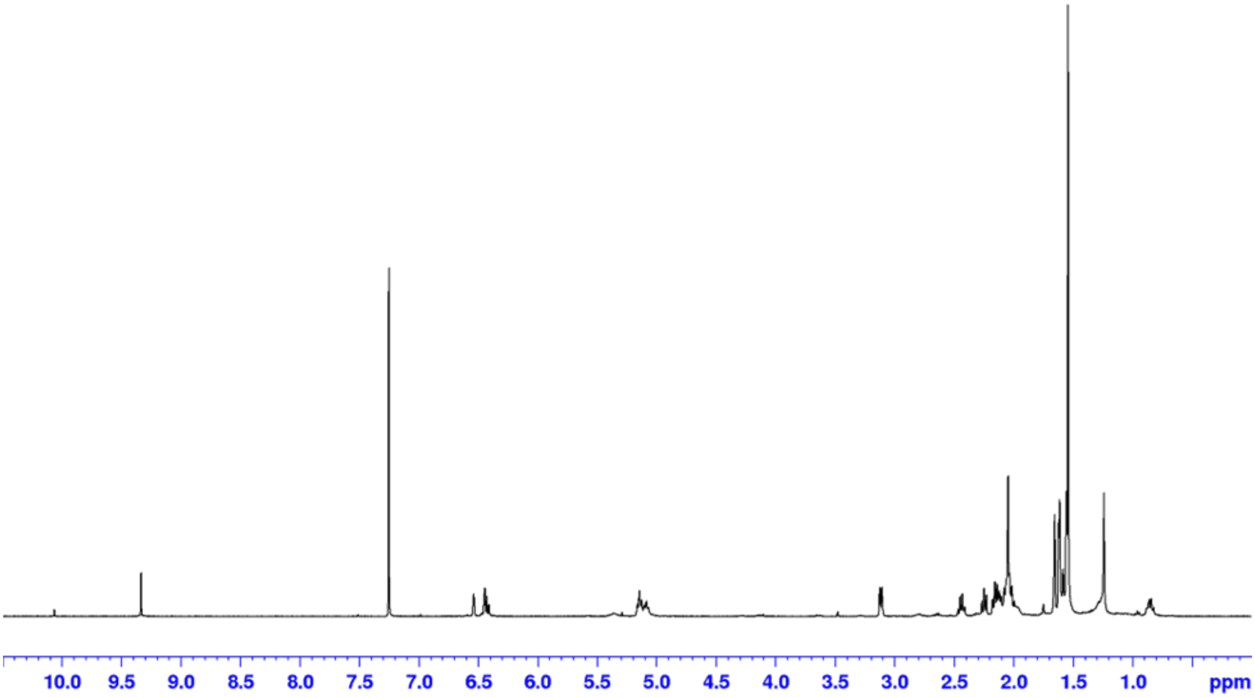

Figure S3. ^{1}HNMR spectrum of compound **2** (Sargaquinal) ($CDCl_3$, 400 MHz).

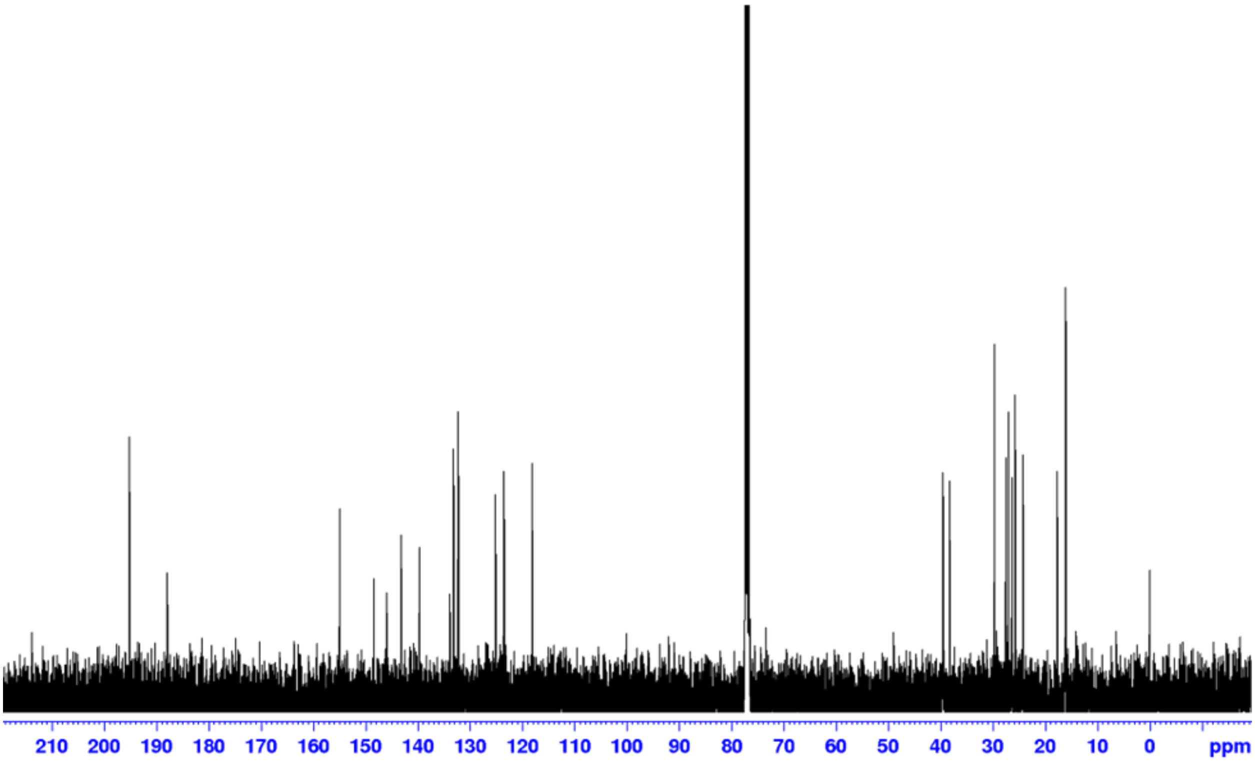

Figure S4. ^{13}C NMR spectrum of compound **2** (Sargaquinal) ($CDCl_3$, 400 MHz).

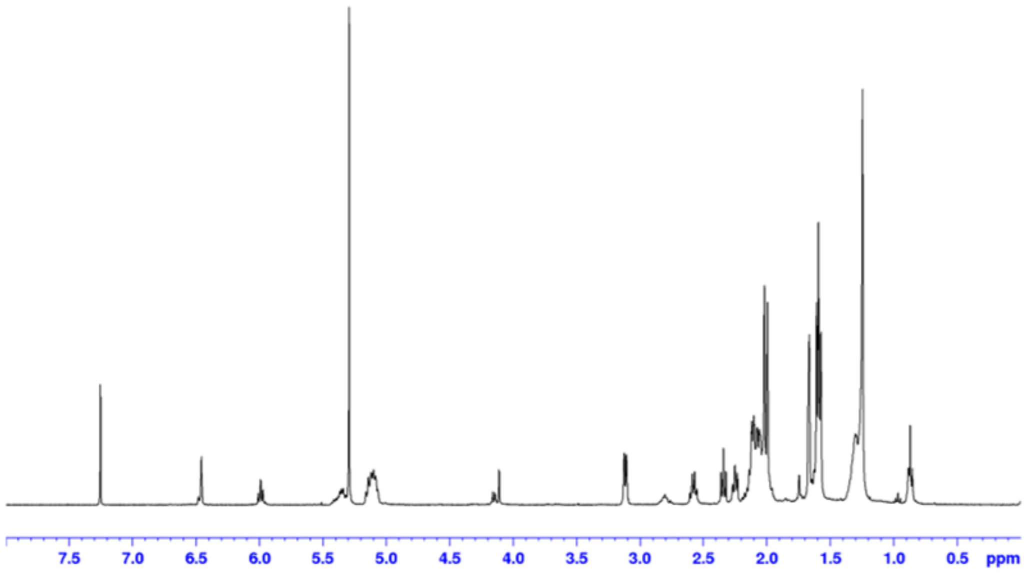

Figure S5. ^{1}H NMR spectrum of compound **3** ($CDCl_3$, 400 MHz).

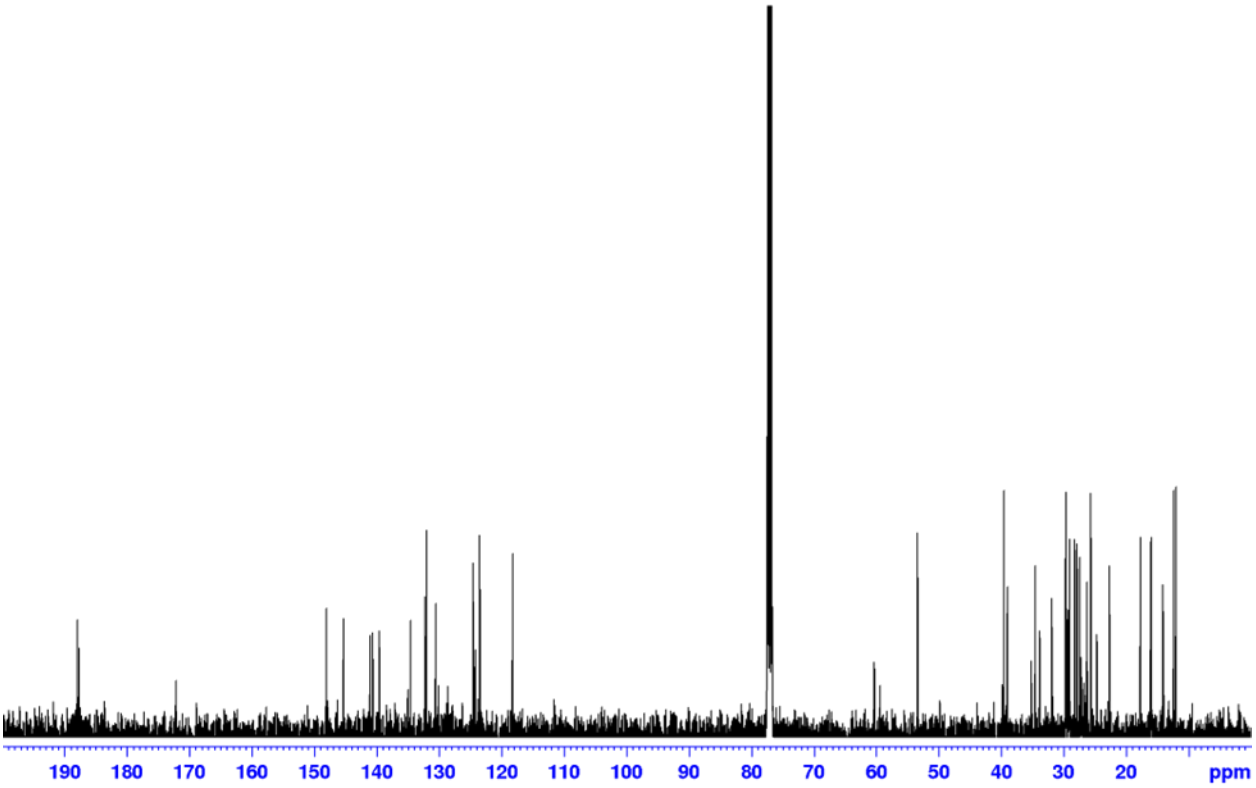

Figure S6. ^{13}C NMR spectrum of compound **3** ($CDCl_3$, 400 MHz).

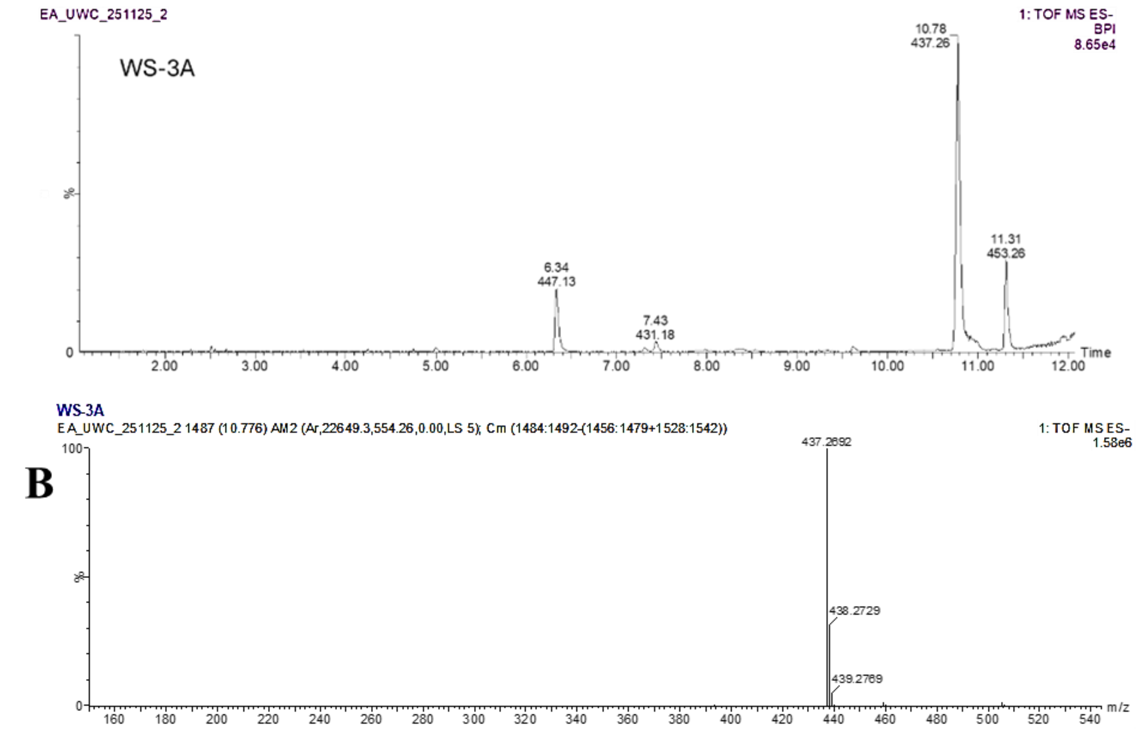

Figure S7. LC-HRMS data obtained for compound **3** showing A) the total ion chromatogram (TIC) and B) the mass spectrum of the peak eluting at 10.776 min.

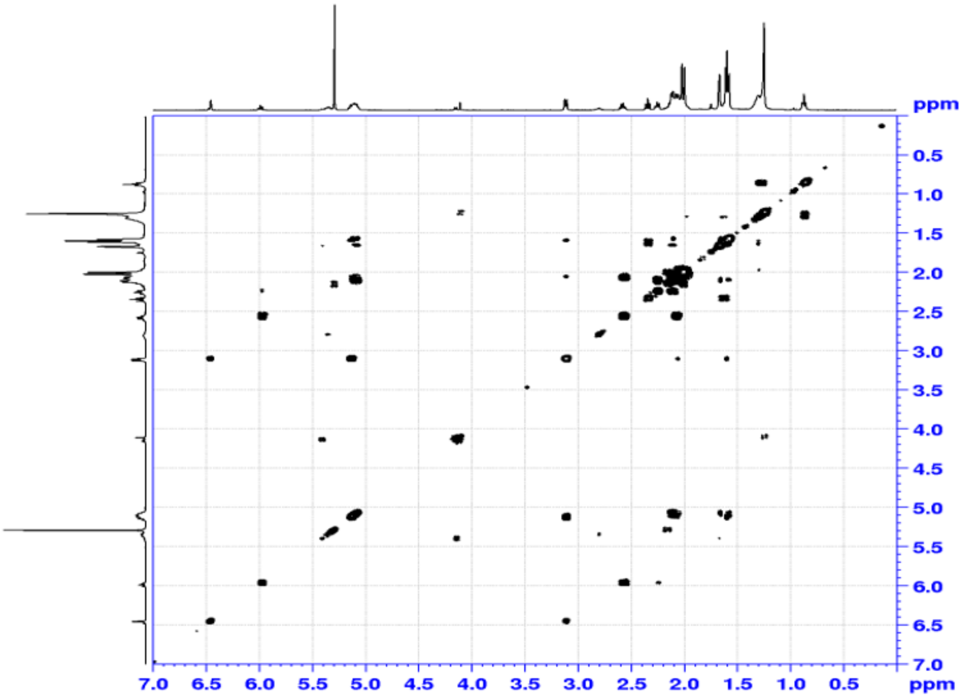

Figure S8. COSY spectrum (400 MHz, $CDCl_3$) of compound **3** (3-methyl sargaquinoic acid).

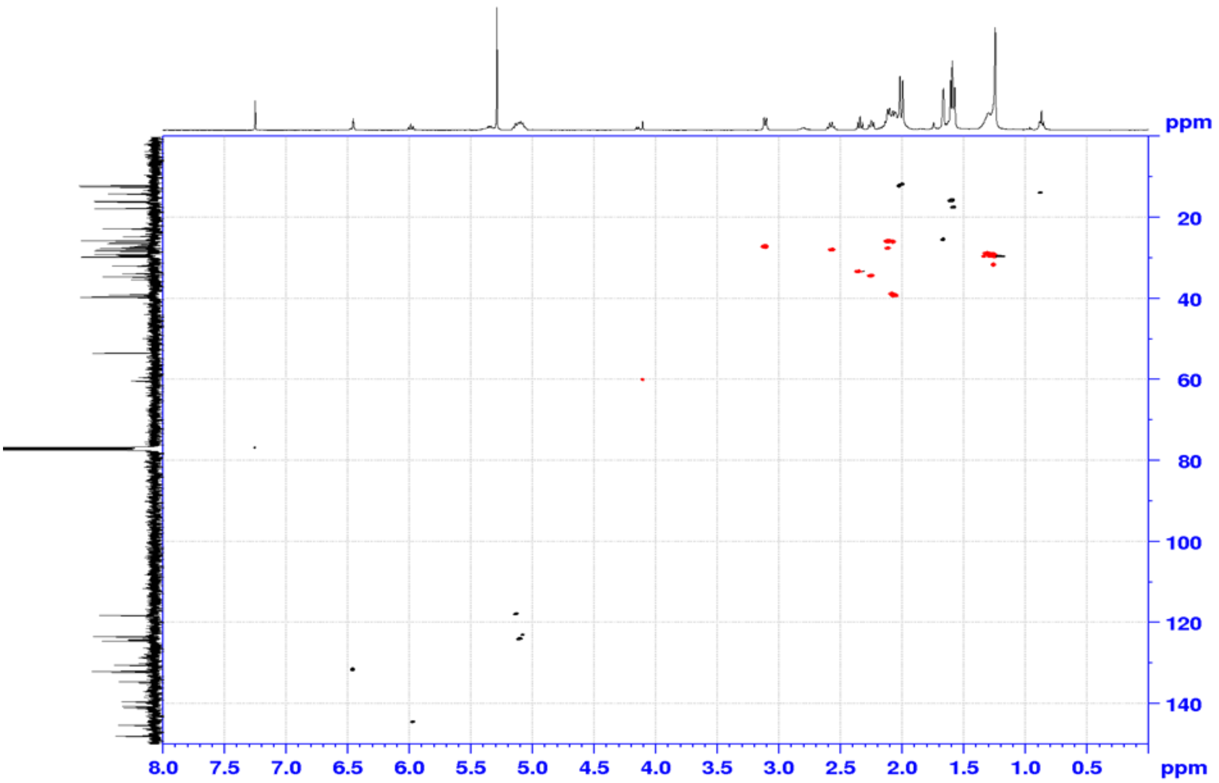

Figure S9. HSQC spectrum (400 MHz, $CDCl_3$) of compound **3 (**3-methyl sargaquinoic acid).

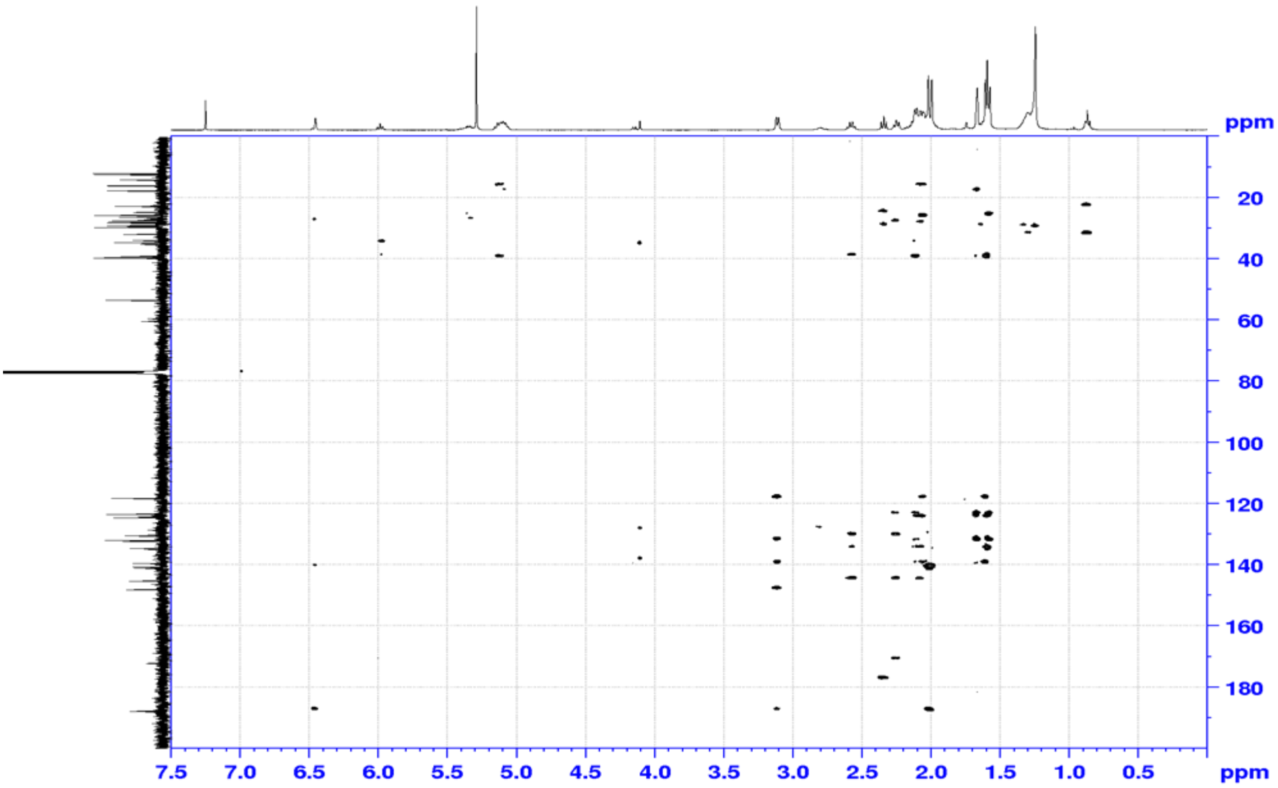

Figure S10. HMBC spectrum (400 MHz, $CDCl_3$) of compound **3 (**3-methyl sargaquinoic acid).

Table S1. ^{1}H (400 MHz), ^{13}C (100 MHz) NMR and 2D NMR (COSY, HSQC, HMBC) spectroscopic data for compound **3**.

**Carbon #**	**d_C (mult.)**	**d_H (mult., J_{Hz})**	**COSY**	**HMBC**	**NOESY**	**TOCSY**
1	187.9 (C)	-	-	-	-	-
2	141.0 (C)	-	-	-	-	-
3	140.6 (C)	-	-	-	-	-
4	187.7 (C)	-	-	-	-	-
5	132.0 (CH)	6.46 (s)	H1′	C3, C4, C1′		H1′, H2′
6	148.0 (C)	-	-	-	-	-
7	12.0 (CH_3)	1.99 (m)	-	C1, C2	-	-
8	12.4 (CH_3)	2.02 (m)	-	C2, C4	-	-
1′	27.4 (CH_2)	3.12 (d, 7.1)	H5, H2′	C2, C4, C5, C2′, C3′	**H5**, H2′, H20′	H5, H2′, H4′, H20′
2′	118.2 (CH)	5.14 (m)	H1′	C4′, C20′	H4′	H1′
3′	139.5 (C)	-	-	-	-	-
4′	39.6 (CH_2)	2.05 (m)*	H5′,	C2′, C3′, C6′,		-
5′	27.8 (CH_2)	2.11 (m)*	-	C3′, C4′, C6′		-
6′	124.5 (CH)	5.10 (m)	H5′	C4′	H5′	-
7′	134.6 (C)	-	-	-	-	-
8′	39.0 (CH_2)	2.07 (m)*	-	C7′, C10′	H12′	-
9′	28.2 (CH_2)	2.57 (q, 7.4)	H8′, H10′	C7′, C8′, C10′, C11′		H8′, H10′
10′	145.3 (CH)	5.98 (t, 7.3)	H9′, H12′	C3, C8′, C12′		H8′, H9′
11′	130.5 (C)	-	-	-	-	-
12′	34.6 (CH_2)	2.25 (t, 7.1)	H13′	C3, C10′, C11′, C14′		-
13′	26.3 (CH_2)	2.10 (m)*	H12′, H14′	-		-
14′	123.4 (CH)	5.08 (m)	H13′	C17′		H12′
15′	132.2 (C)	-	-	-	-	-
16′	25.6 (CH_3)	1.68 (m)	-	C14′, C15′, C17′		-
17′	17.7 (CH_3)	1.58 (m)	-	C14′, C15′, C16′		-
18′	172.1 (C)	-	-	-	-	-
19′	16.1 (CH_3)	1.60 (m)	-	C6′, C7		-
20′	15.9 (CH_3)	1.61 (m)	H2′	C2′, C3′		-

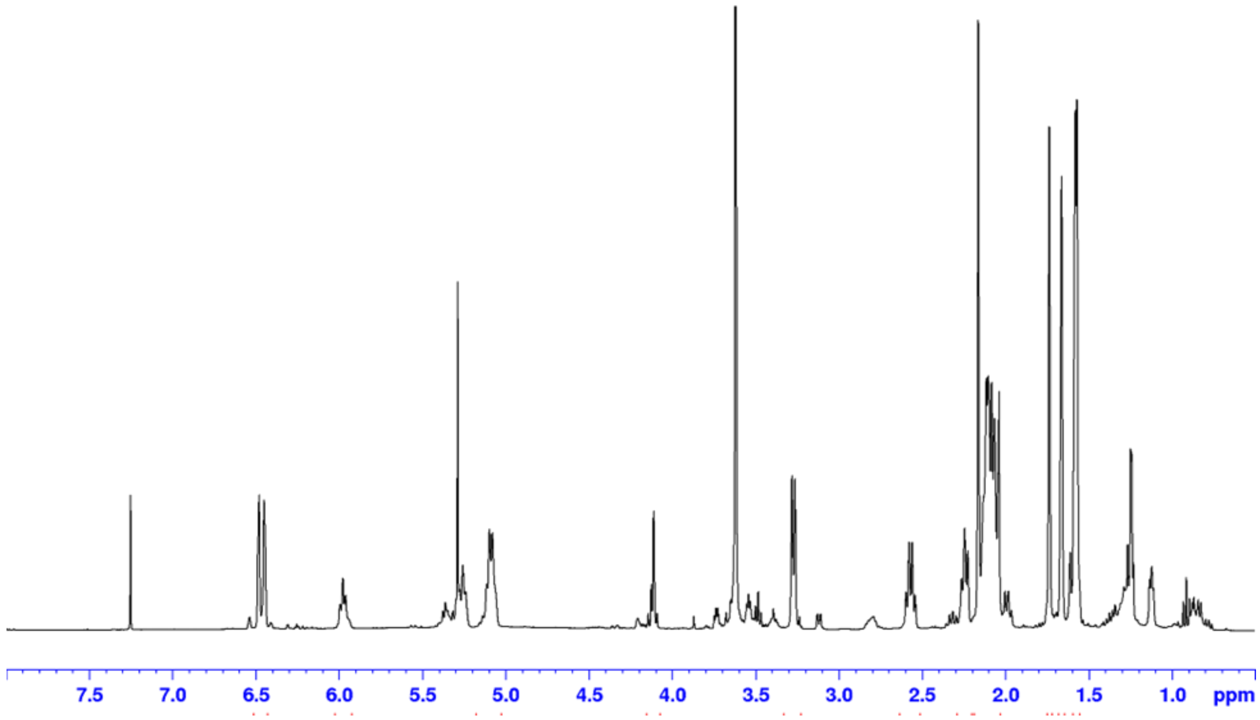

Figure S11. ^{1}H NMR spectrum of compound **4** (Sargahydroquinoic acid) ($CDCl_3$, 400 MHz).

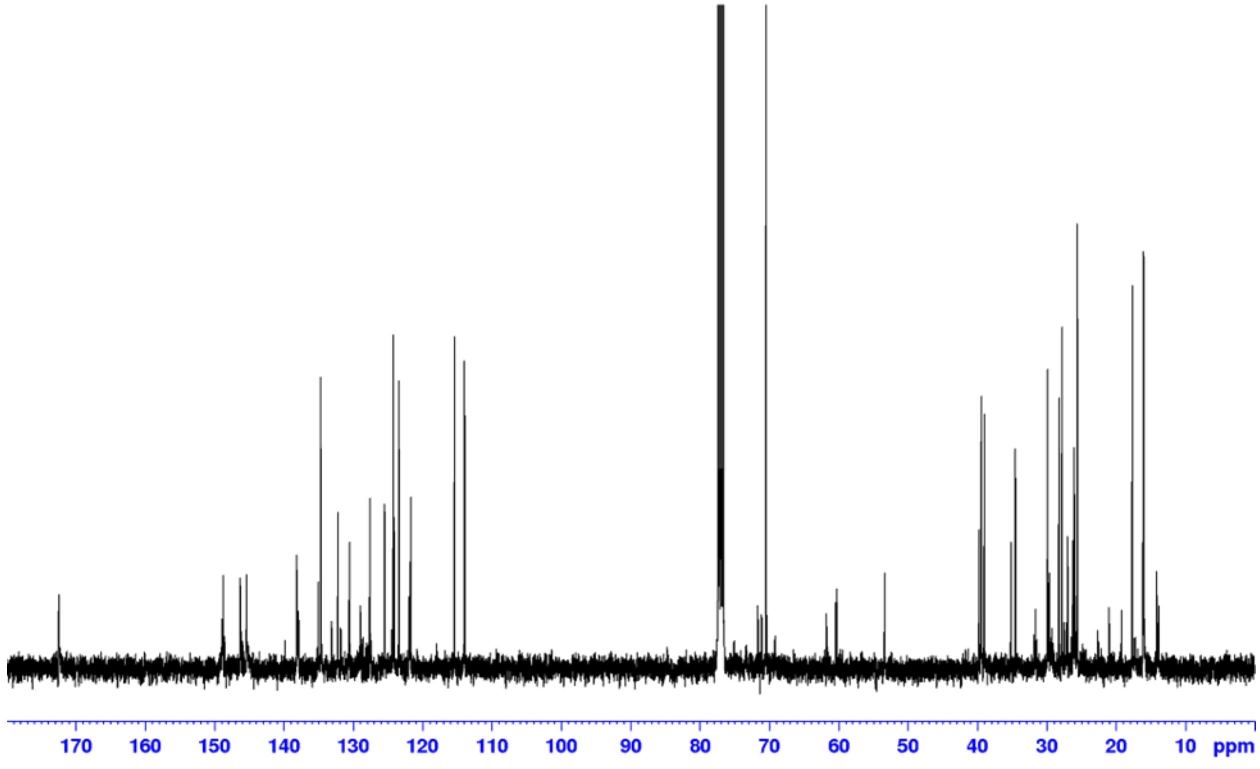

Figure S12. ^{13}C NMR spectrum of compound **4** (Sargahydroquinoic acid) ($CDCl_3$, 100 MHz).

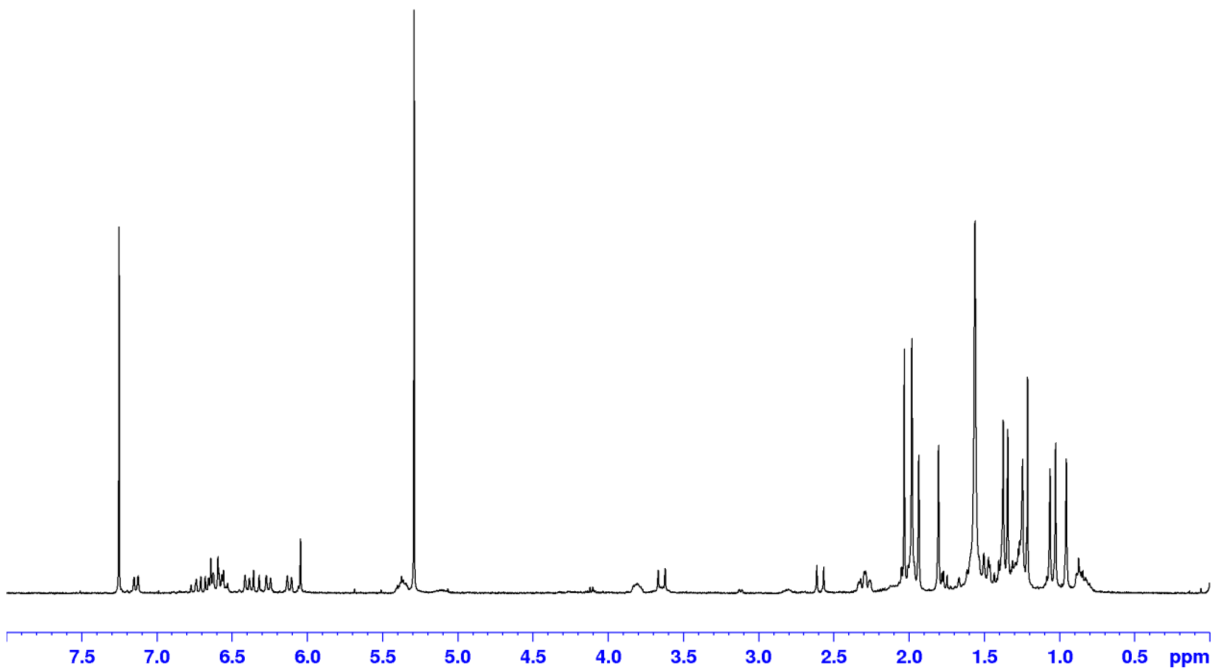

Figure S13. ^{1}H NMR spectrum of compound **5** (Fucoxanthin) ($CDCl_3$, 400 MHz).

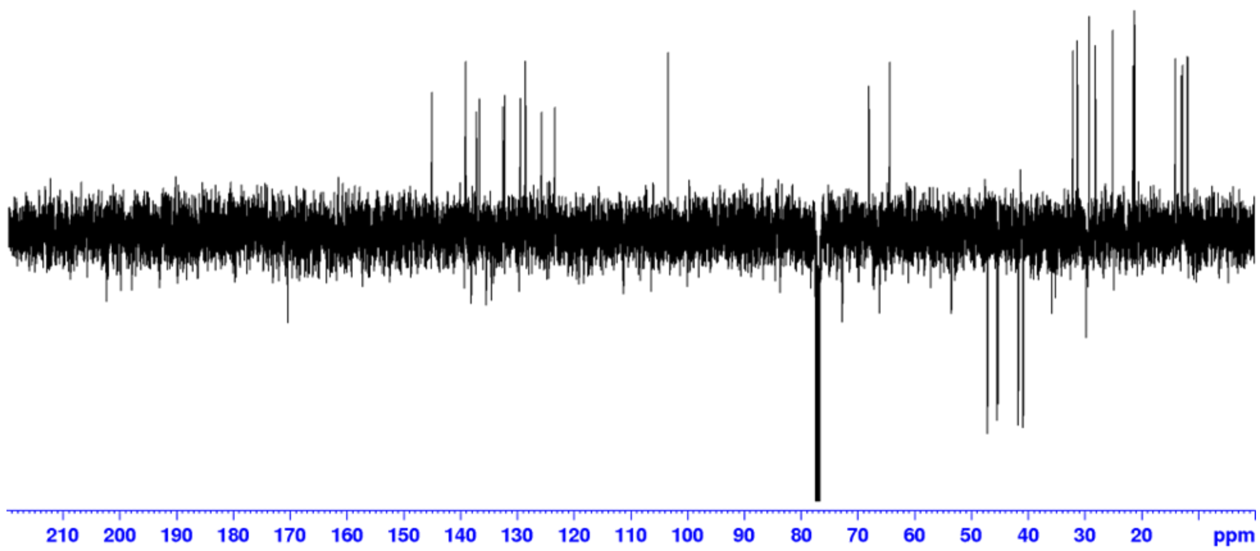

Figure S14. ^{13}C DEPTQ-135 NMR spectrum for compound **5** (Fucoxanthin) ($CDCl_3$, 100 MHz).

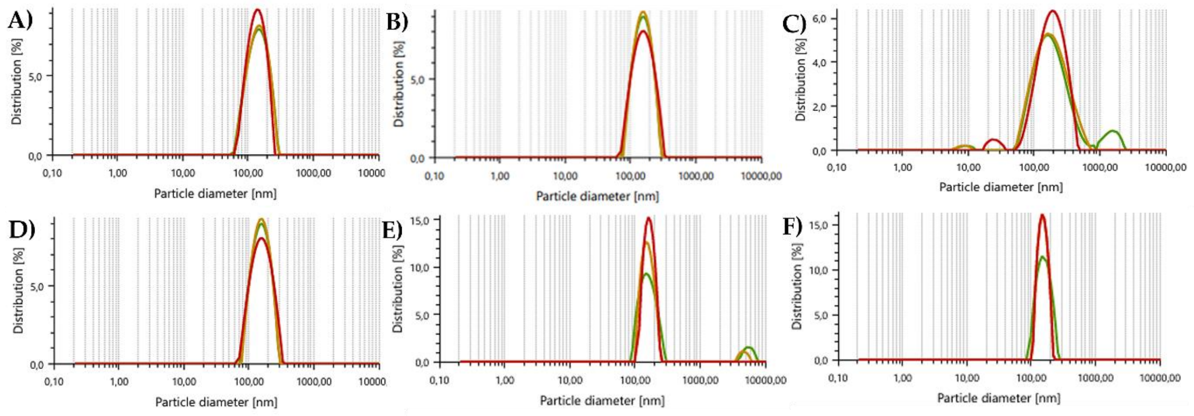

Figure S15. Dynamic light scattering (DLS) size distribution profiles of solid lipid nanoparticles (SLNs): (a) blank SLNs, (b) tocopherol-loaded SLNs, (c) crude extract-loaded SLNs, (d) sargaquinoic acid (SQA, **1**)-loaded SLNs, (e) sargahydroquinoic acid (SHQA, **4**)-loaded SLNs, and (f) fucoxanthin (FX, **5**)-loaded SLNs. The measurements represent the hydrodynamic diameter distributions of the nanoparticles in aqueous suspension, confirming nanoscale particle sizes and relatively narrow size distributions across all formulations.

Table S2. Stability of solid lipid nanoparticles (SLNs) in Nutrient Broth (NB) measured at 0, 1, and 24 hours using DLS. The table shows changes in particle size, PDI, and zeta potential over time.

Sample	Media	Hydrodynamic Radii (d, nm)	Polydispersity Index	Zeta potential (mV)
**t = 0**	Nutrient broth	197.89 ± 1.12	0.170	-6.27 ± 2.73
	Tryptic soy broth	193.68 ± 5.18	0.132	-8.17 ± 1.80
	Yeast malt	208.80 ± 5.07	0.169	-6.37 ± 0.85
**t = 1hr**	Nutrient broth	208.00 ± 0.94	0.160	-10.4 ± 1.22
	Tryptic soy broth	214.67 ± 1.10	0.139	-13.83 ± 0.70
	Yeast malt	219.13 ± 0.85	0.162	-8.00 ± 0.20
**t = 24 hrs**	Nutrient broth	232.77 ± 5.22	0.118	-10.8 ± 0.80
	Tryptic soy broth	255.53 ± 4.20	0.153	-10.37 ± 0.80
	Yeast malt	269.90 ± 3.70	0.149	-13.83 ± 0.70

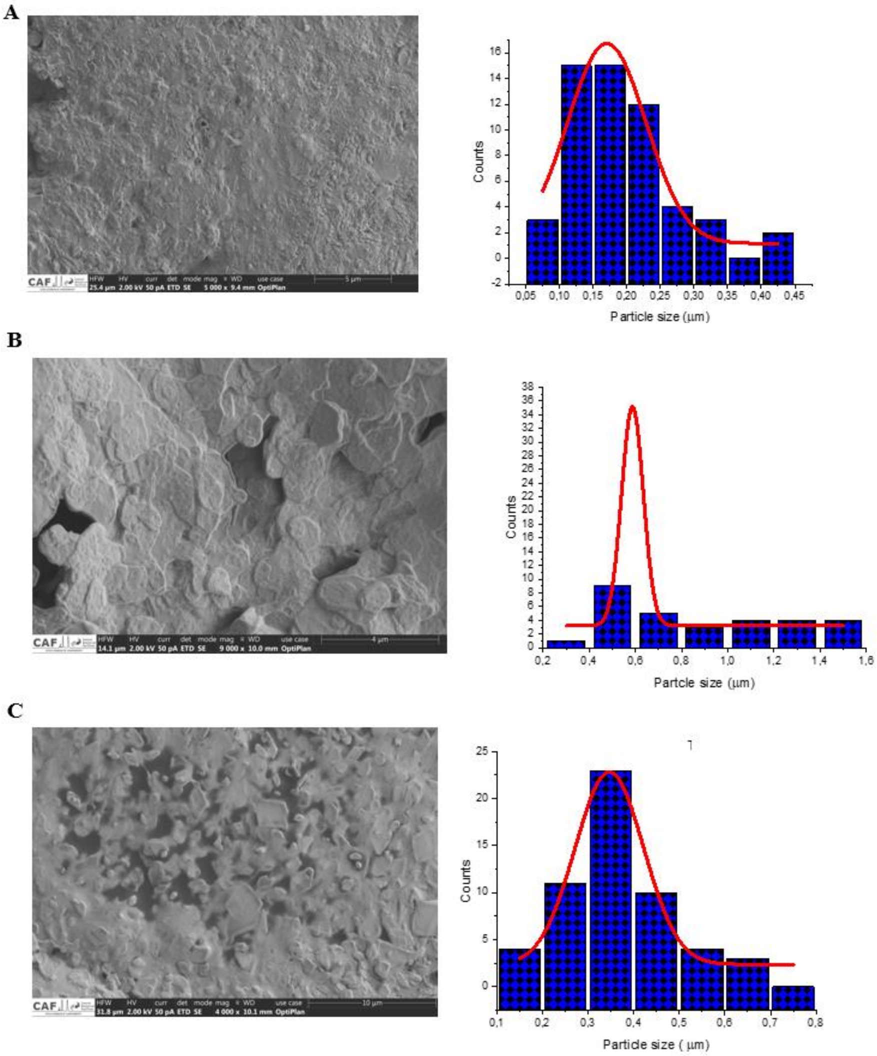

Figure S16. SEM images and particle size distribution histograms of the SLN formulations for (A) Blank SLNs, (B) Crude-SLNs (*S. incisifolium* crude extract), and (C) Tocopherol-SLNs. Scale: 50 µm.

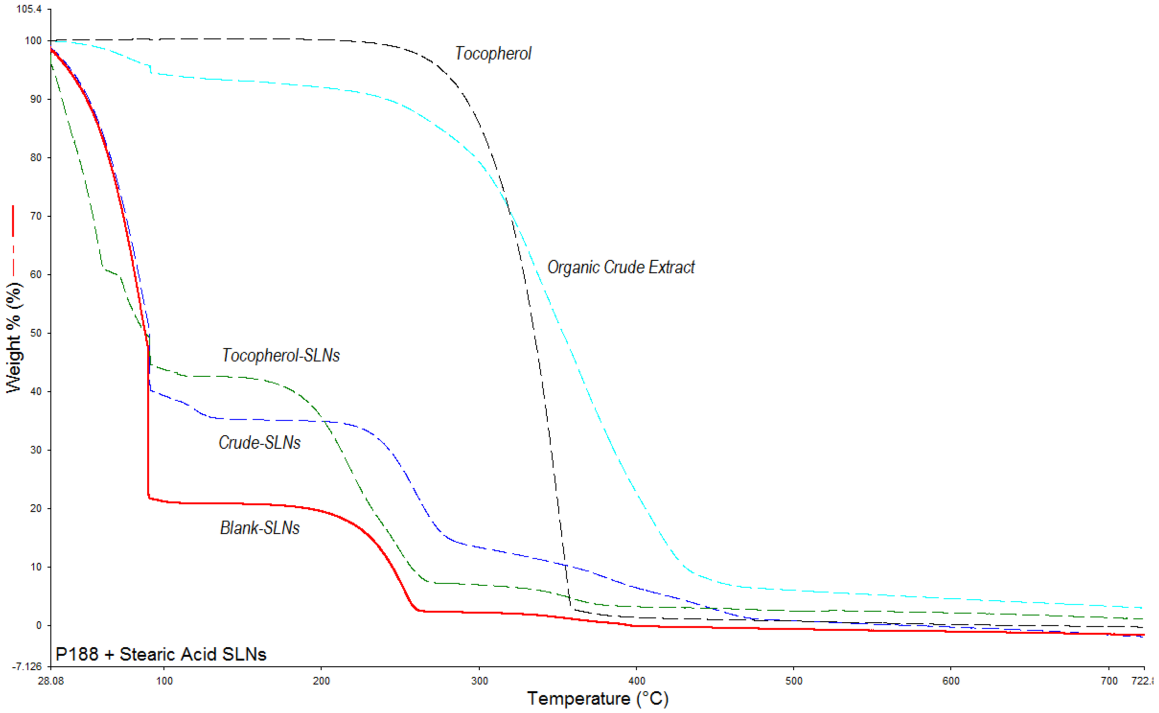

Figure S17. TGA profiles for α-tocopherol (black), the crude extract (light blue), blank SLNs (red), and a-tocopherol loaded SLNs (green), and crude extract loaded SLNs (blue).

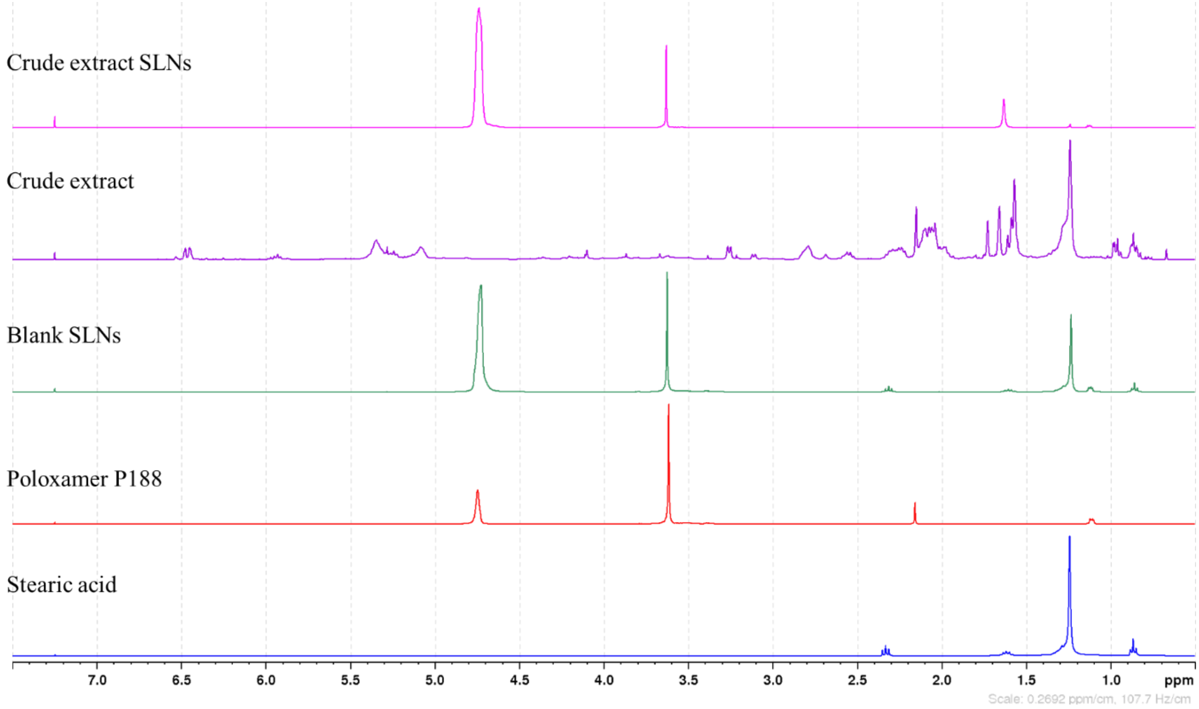

Figure S18. ^{1}H NMR spectra (400 MHz, $CDCl_3$) of stearic acid, Poloxamer P188, blank SLNs, crude organic extract, and the crude organic extract-loaded SLNs showing the incorporation of the components into the SLN formulation.

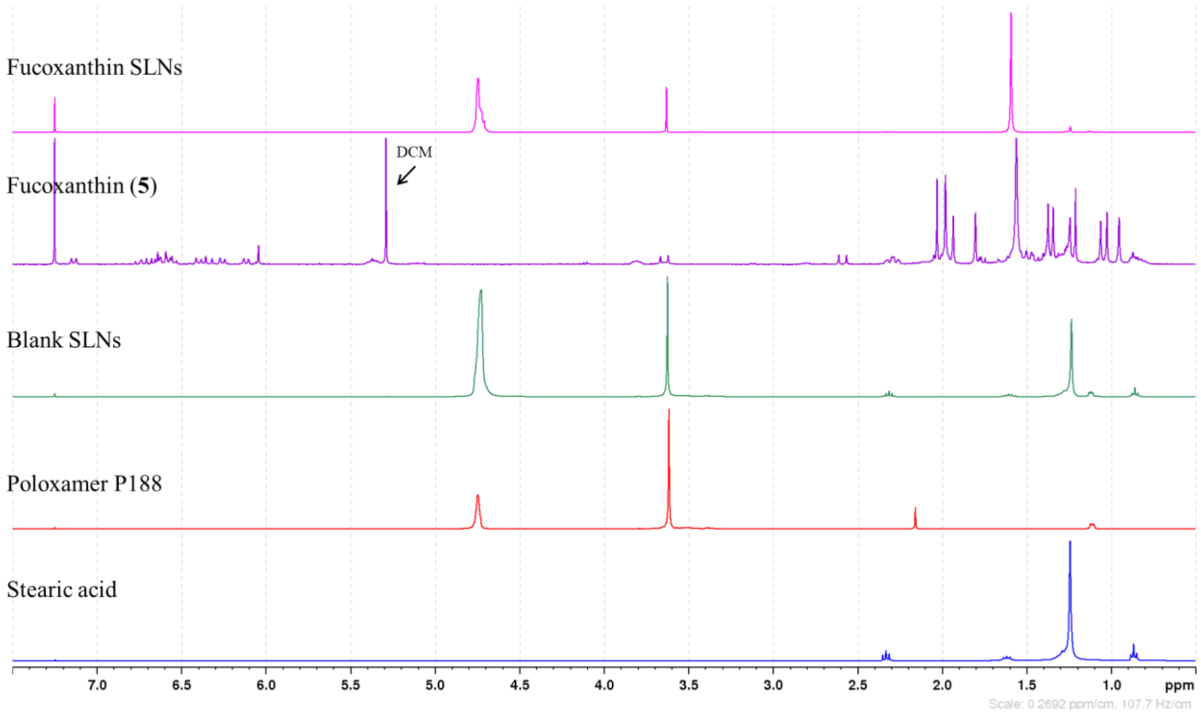

Figure S19. ^{1}H NMR spectra (400 MHz, $CDCl_3$) of stearic acid, Poloxamer P188, blank SLNs, fucoxanthin (**5**), and the fucoxanthin (**5**)-loaded SLNs showing the incorporation of the components into the SLN formulation.
